# Occlusal Fissures in Equine Cheek Teeth: A Prospective Longitudinal *in vivo* Study

**DOI:** 10.3389/fvets.2020.604420

**Published:** 2020-11-17

**Authors:** Elke Pollaris, Bart J.G. Broeckx, Lieven Vlaminck

**Affiliations:** ^1^Faculty of Veterinary Medicine, Department of Surgery and Anaesthesiology of Domestic Animals, Ghent University, Merelbeke, Belgium; ^2^Faculty of Veterinary Medicine, Department of Nutrition, Genetics and Ethology, Ghent University, Merelbeke, Belgium

**Keywords:** crown fracture, cheek teeth, equine dentistry, idiopathic cheek teeth fractures, fissure

## Abstract

**Background:** It has been suggested that fissures of the occlusal surface of equine cheek teeth may develop into crown fractures.

**Objectives:** To examine the evolution of fissures present on the occlusal surface of cheek teeth. Furthermore, to investigate the presence of a fissure as a risk factor for the development of a subsequent crown fracture.

**Study Design:** Observational longitudinal study.

**Methods:** Bi-annual dental examinations were performed on 36 horses for 3 years. Video-recordings were made to evaluate the evolution of detected fissures. The effect of possible predictors on the development of tooth fractures was investigated by regression analysis.

**Results:** The evolution of 785 fissures (467 type 1a, 271 type 1b, 47 type 2) was recorded. Fissure characteristics were observed to remain unchanged, disappear, become longer, shorter, change in configuration or change in color. Partial crown fractures (22 maxillary, 50 mandibular) were recorded in 52 cheek teeth in 22/36 horses. Fifty-nine of these fractures evolved from previously observed fissures (24 type 1a, 29 type 1b, 6 type 2). All fissure types proved to be a significant risk factor for the development of a crown fracture (*p* < 0.001), with the highest odds for type 2 fissures (*OR* = 14.27; 95% CI = 4.88–41.71). Other significant risk factors were the time of follow-up (*p* < 0.001), mandibular teeth (*p* < 0.001) and the lingual side of a tooth (*p* < 0.001). All fractures were non-complicated.

**Main Limitations:** Some horses were prematurely lost for follow-up, which perhaps influenced the results. A longer follow-up period would have also allowed an evaluation of the risk for pulp disease on the long term subsequent to partial crown fractures.

**Conclusions:** The presence of a fissure of any type, mandibular cheek teeth, the lingual side of cheek teeth, and time of follow-up proved to be significant risk factors for development of a cheek tooth crown fracture. Type 2 fissures showed the highest odds followed by type 1b fissures. The observed partial crown fractures demonstrated a low clinical impact whereby no tooth showed signs of development of endodontal disease.

## Introduction

Occlusal fissures, a common finding in equine cheek teeth during dental examination, are generally considered innocuous (1–3). However, in a previous study pulpitis was considered to be caused through existing occlusal fissures in 2 out of 79 (2.5%) apically infected cheek teeth (4). Oral bacteria might invade the space that is created by a fissure and colonize pulp tissue as was demonstrated by Wellmann and Dixon (5), and further supported by the findings of Pollaris et al. (6). However, it was also concluded that healthy pulp should be able to deal with these noxious stimuli (6).

Fissures have been suggested to be the early stage of crown fractures (3, 4, 7). Specifically, some fissure configurations that correspond to well-known fracture configurations (e.g., some type 1b fissures according to the classification by Pollaris et al., correspond to buccal slab fractures) (3, 8) are hypothesized to be more at risk to evolve toward a crown fracture. Ramzan and Palmer (2) stated that fissures with a largely transverse orientation [type 1a (3)] are not likely to develop into gross fractures. To the authors' knowledge, no papers have been published that studied the *in vivo* progression of fissures.

The aim of this study is to examine the evolution of fissures in time. We hypothesized that fissures may be seen as the preliminary stage for later crown fracture and that some fissure configurations are more prone to progress toward a crown fracture.

## Materials and Methods

### Study Design

The experimental protocol was approved by the Ghent University Committee on the care and use of experimental animals in compliance with the Belgian legislation on animal experiments. Every 6 months for a period of 3 years (T0-6), a thorough dental examination was performed by the same person (EP) on each horse of a faculty-owned herd. This examination was performed on the sedated horse (detomidine; Detogesic, 15 μg/kg bwt, IV and butorphanol; Torbugesic, 15 μg/kg bwt, IV), using a full mouth speculum and an oral endoscope. Video recordings were made for retrospective analysis. The presence or absence of occlusal fissures as well as specific fissure characteristics (position of the fissure, fissure type, configuration, color) were recorded for every individual cheek tooth. Fissures were classified according to Pollaris et al. (3) as:

- Type 1a fissure: involves the secondary dentine overlying the pulp cavity and runs from the secondary dentine perpendicular to the surrounding enamel fold.
- Type 1b fissure: involves the secondary dentine overlying the pulp cavity but does not follow a perpendicular orientation in relation to one surrounding enamel fold. Often this orientation is more mesio-distal.
- Type 2 fissure: does not involve secondary dentine.

Type 2 fissures were only recorded when they involved primary dentin. Small enamel cracks which were commonly observed in a cadaveric study (3) were not included due to the difficulty to observe them on video recordings and their minimal clinical importance as has been demonstrated in a high-resolution X-ray computed tomography study (6). The development of crown fractures was recorded during the follow-up period and their relationship with previously identified fissures was noted.

Treatment of encountered dental pathology was only performed when associated with signs of oral discomfort (e.g., soft tissue lesions, periodontal disease, etc.). Prophylactic odontoplasty was not performed and the occlusion of the cheek teeth was not changed in the absence of any masticatory problem.

### Experimental Animals

Thirty-six horses were included in the study (university-owned herd), none of which had any known dental history. All horses were housed under similar conditions. Whenever possible, they were kept on pasture or otherwise stall-rested in separate boxes. As long as pasture quality allowed this, horses were not given any extras when being kept outside. When housed indoors, they were fed 1 kg of concentrates twice daily and were given hay *ad libitum*.

### Statistical Analysis

Data were recorded on a spreadsheet for descriptive statistics. Chi-square tests were used in intergroup comparisons of categorical variables (maxilla/mandible vs. fissure characteristics; fissure type vs. fissure characteristics). Categorical variables were expressed as numbers and percentages. Continuous variables were presented as mean ± standard deviation (and range, when interesting).

The effect of possible predictors on the development of tooth fractures was analyzed. The overall significance was set at α ≤ 0.05. Firstly, the effect of individual predictors (time of follow-up, gender (male/female), age, dental pathology (no pathology, wear disorder, periodontitis), maxilla/mandible, tooth (second premolar—third molar), side of the tooth (lingual/buccal), fissure present (yes/no), fissure type (no fissure, type 1a, type 1b, type 2) on the development of fractures (dependent variable, yes/no) was assessed with a generalized linear mixed model with the individual predictor as fixed effect and horse as random effect. Significance was assessed with a likelihood ratio test. *P*-values were corrected for multiple testing by multiplying them by the number of tests and are reported as such. Next, the most optimal generalized mixed model was determined based on the combination of predictors that minimized the Akaike Information Criterion. The program R version 3.5.2 (“Eggshell Igloo”) was used for statistical analyses (9).

## Results

Thirty-six horses (864 teeth) were included in the study at the start of the observational period (T0), including 20 mares, 14 geldings, and 2 stallions. The mean age of the horses at T0 was 12.6 (±4.55) years. The mean duration of follow-up of the horses was 2.32 (±0.98) year. Follow-up for the complete duration of the study (3 years) was possible in 22/36 animals. Follow-up records of 14/36 horses were incomplete due to reasons unrelated to this study (euthanasia, adoption). From these horses, 6 could be followed for 6 months, 2 for 12 months, 3 for 18 months, 2 for 2 years, and 1 for 2.5 years. At T0, 14/36 horses showed no dental pathology, 15/36 horses had wear disorders [wave mouth (*n* = 10), mandibular distoclusion (*n* = 3), both (*n* = 2)], and 7/36 horses had periodontal disease (2 were quidding). Horses with periodontal disease were treated with diastema debridement, corrective odontoplasty, grooving of the interproximal space and diastema occlusion with polyvinylsiloxane. During the study period, 5 teeth were extracted due to progressive periodontal disease. In 9 horses, an uncomplicated crown fracture (an enamel-dentin fracture not involving the pulp horn) was recorded at T0 in 13 teeth. In 1 horse a complicated crown fracture was present with an exposed pulp cavity [left mandibular third premolar tooth (307), partial crown fracture at the level of pulp horn 5]. This tooth showed signs of a chronic apical infection on radiographic examination and was subsequently extracted.

### General Fissure Observations

In 34/36 horses, fissures were observed at T0 with a mean of 15.64 (±15.05; range: 0–69) fissures per horse. At the start of the study (T0), 563 fissures were recorded (357 type 1a, 186 type 1b and 20 type 2) whereas 271 new fissures appeared over time (137 type 1a, 101 type 1b and 33 type 2) (Supplementary Information 1).

**Figure 1:**
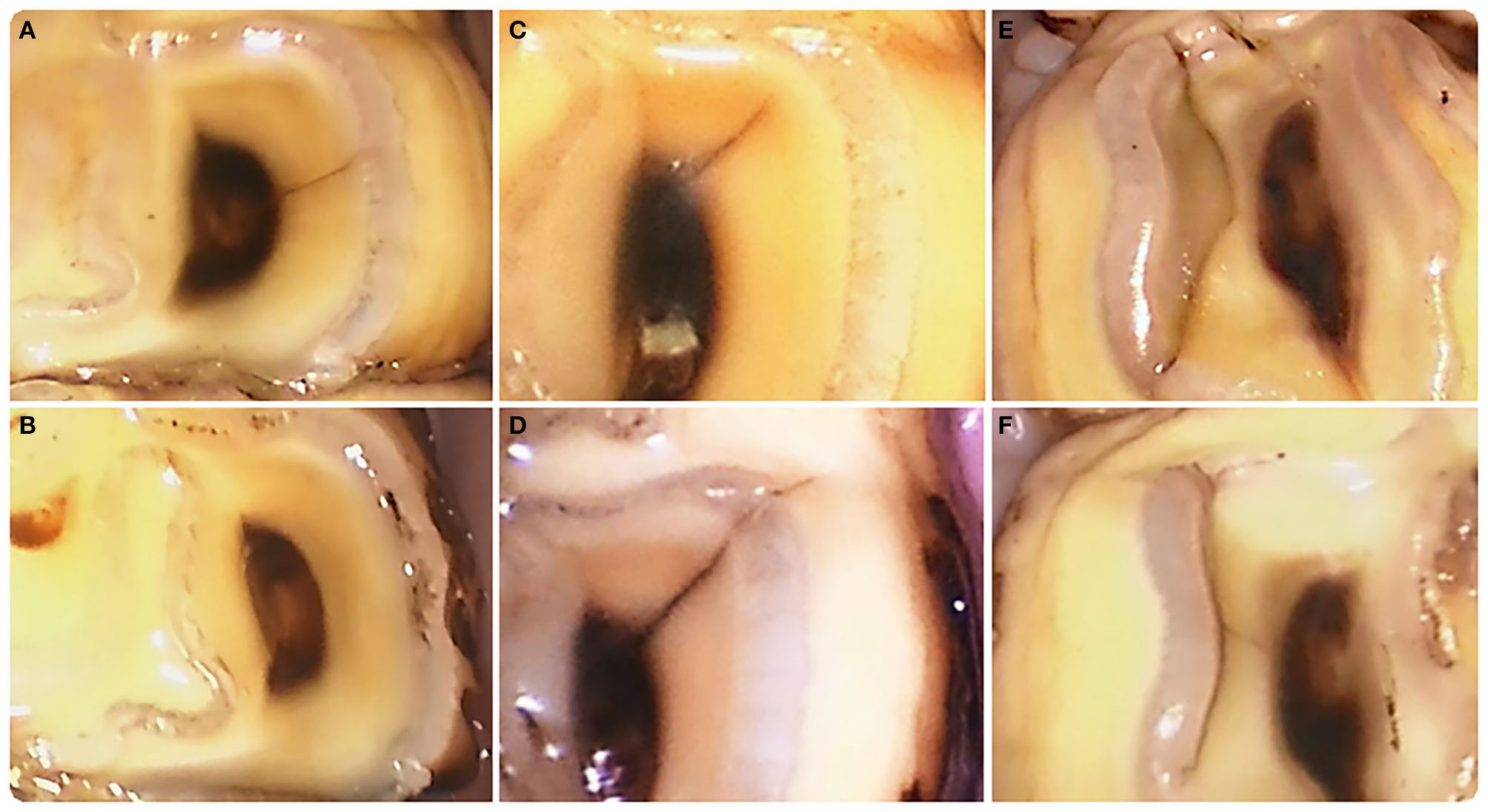
Examples of fissure evolution in time. **(A)** Type 1a fissure at pulp horn 1 in a left mandibular first molar tooth (309) (T0). **(B)** This fissure had disappeared after 3 years (T6). **(C)** Type 1a fissure at pulp horn 1 in a left mandibular second molar tooth (310) (T0). **(D)** This fissure became longer after 1 year (T2) and developed into a crown fracture after 2.5 years (T5) (Figures 3A,B). **(E)** Type 2 fissure near the secondary dentine of pulp horn 2 in a right mandibular third premolar tooth (407) (T0). **(F)** Two and a half years later (T5) the fissure location was closer to the enamel ring. ***(A)****is top left*, ***(B)****is bottom left*, ***(C)****is top middle*, ***(D)****is bottom middle*, ***(E)****is right top, and* ***(F)****is bottom right*.

**Figure 2:**
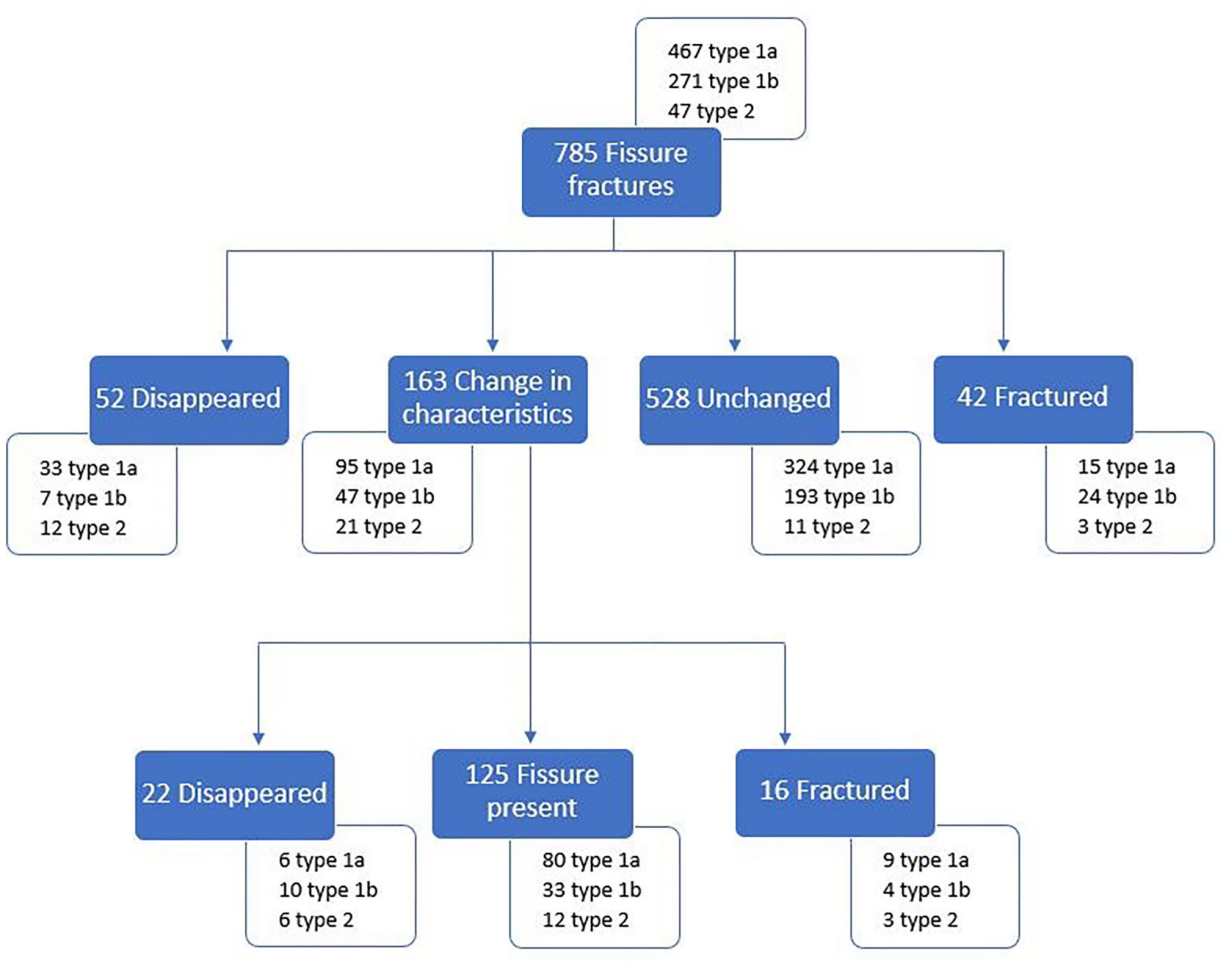
Flow chart illustrating an overview of the evolution of the observed fissures.

During the study, the evolution of 785 fissures could be followed of which 381 were present on maxillary cheek teeth (182 type 1a, 176 type 1b, and 23 type 2) and 404 on mandibular cheek teeth (285 type 1a, 95 type 1b, 24 type 2). Fissures were observed to remain unchanged, disappear, become longer or shorter, change in configuration (shape and/or slight positional change) or change in color (Figure 1). Overall, fissures remained unchanged in 67.3% (528/785), disappeared in 9.4% (74/785) and changed in length/configuration/ color in 20.8% (163/785) while the remainder fractured (Figure 2). The average time after which fissures disappeared after their first observation was 1.62 (±0.88) year. The fissure length increased in 7.5% (59/785) and decreased in 4.7% (37/785) of cases. Configuration changes were recorded in 5.2% (41/785). Fissure staining became darker in 3.1% (24/785) and lighter in 2.4% (19/785). A detailed overview of the distribution of these fissures can be found in Supplementary Information 2.

Fissure evolution was different in mandibular compared to maxillary cheek teeth, however only significantly for fissures that remained unchanged. In the maxilla, 72.4% of the fissures remained unchanged whereas in the mandible this was 62.4% (*p* = 0.003). Fissures changed in length/configuration/color in 18.1% in the maxilla and 23.3% of the mandible (*p* = 0. 075). The number of fissures that disappeared was relatively similar in maxillary (10.2%) and mandibular (8.7%) teeth (*p* = 0.45). Fissure evolution was also different between fissure types. Type 2 fissures changed in length/configuration/color more frequently (44.7%, 21/47) compared to type 1a (20.3%, 95/467) and type 1b (17.3%, 47/271) (*p* < 0.001). The number of fissures that disappeared was significantly higher in type 2 fissures (38.3%, 18/47) (*p* < 0.001). Type 1a and type 1b fissures disappeared in only 8.4% (39/467) and 6.3% (17/271), respectively. An overview of the observations in the maxillary and mandibular arches of fissures that changed in length/configuration/color is given in Supplementary Image 1.

### Crown Fractures

**Figure 3:**
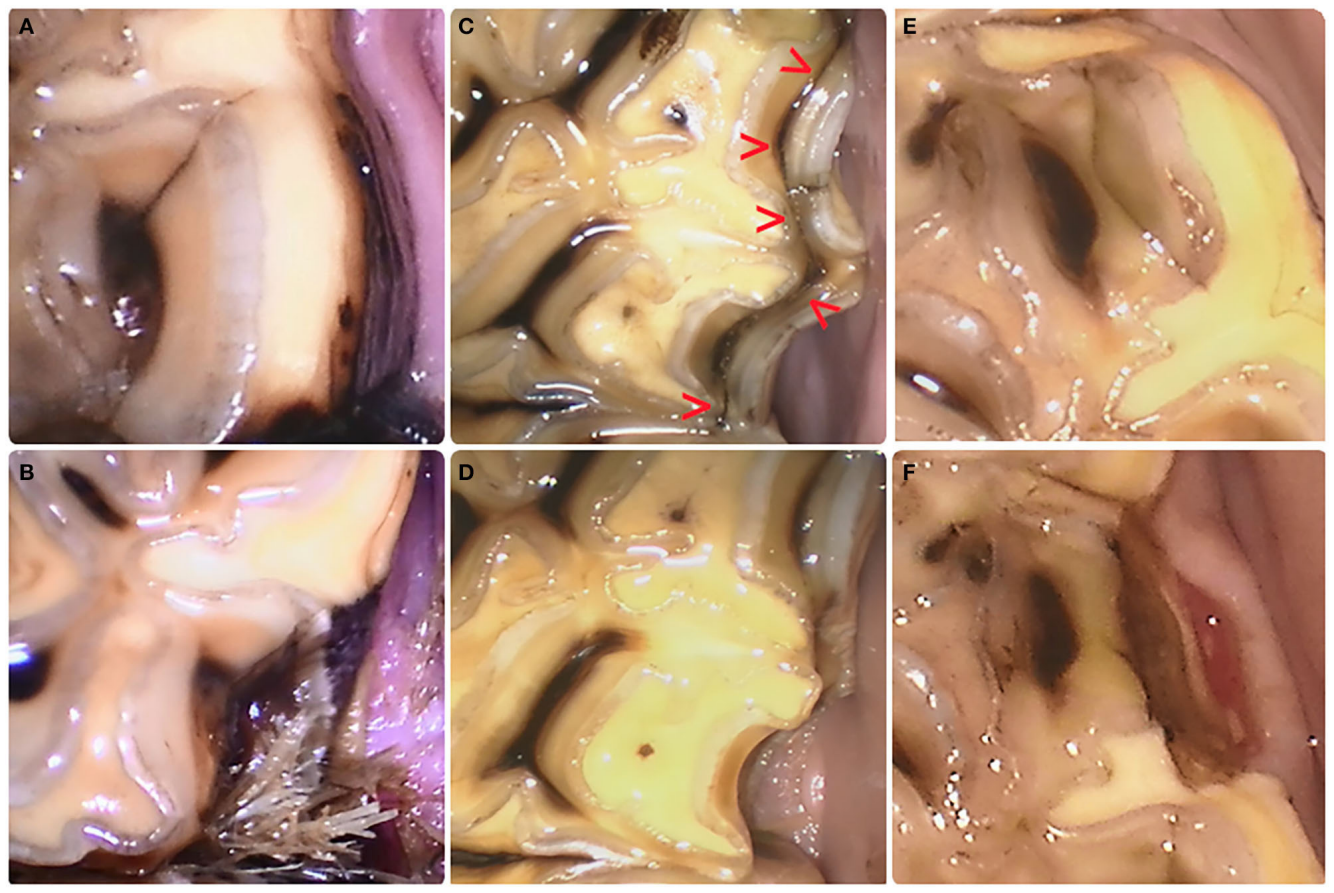
Evolution from fissure to crown fracture. **(A)** Type 1a fissure at the level of pulp horn1 in a left mandibular second molar tooth (310) with subsequent fracture development after 2.5 years. Food impaction between teeth at the fracture site is visible **(B)**. **(C)** This Type 1b fissure (red arrow heads) connecting pulp horn1 and pulp horn2 in a left maxillary fourth premolar tooth (208) evolved toward a partial buccal slab fracture at the level of pulp horn 2 **(D)** after 2.5 years. **(E)** Type 2 fissure located buccal to pulp horn 2 of a left mandibular third premolar tooth (308) resulted in a fracture **(F)** 6 months later. The recent nature of the fracture is evident through the damaged gingival margin. ***(A)****is top left*, ***(B)****is bottom left*, ***(C)****is top middle*, ***(D)****is bottom middle*, ***(E)****is right top, and* ***(F)****is bottom right*.

During the follow-up period, 22/36 horses developed a partial crown fracture in 52 different cheek teeth (18 maxillary and 34 mandibular cheek teeth) with an average of 1.38 (±0.72) fractures per tooth. In total, 72 crown fractures (22 maxillary, 50 mandibular) were observed. Crown fractures in teeth without the previous detection of a fissure were observed in 13 cheek teeth (9 mandibular, 4 maxillary cheek teeth), which were all on the lingual side of the tooth (Supplementary Image 2). Fifty-nine crown fractures originated from a previously identified fissure (24 type 1a, 29 type 1b, and 6 type 2) (Figure 3). One type 1b fissure fractured twice in a different location of the fissure at different time points. Overall, fissuresevolved to a partial crown fracture in 7.4% (58/785). The average time that elapsed between the initial observation of a fissure and the development of a partial crown fracture was 1.36 (±0.91) years. Crown fractures originating from fissures involved maxillary teeth in 18/59 (30.5%) and mandibular teeth in 41/59 (69.5%) of fracture cases. These fractures occurred on the buccal side in 26/59 (44.1%) teeth (7 maxillary, 19 mandibular) and on the lingual side in 33/59 (55.9%) teeth (11 maxillary, 22 mandibular).

The detailed results of the potential association of variable predictors with the presence of a tooth fracture can be found in Table 1. Individual factors that were associated with fracture development included follow-up time (*p* < 0.001), dental pathology (*p* < 0.05), maxilla/mandible (*p* < 0.01), the presence of a fissure (*p* < 0.001), and fissure type (*p* < 0.001). Gender (*p* = 1), Tooth type (*p* = 0.08), fissure on the buccal or lingual side of the tooth (*p* = 0.07), and age of the horse (*p* = 1) were not found to be significant. After construction of the final model, time of follow-up (*p* < 0.001), maxilla/mandible (*p* < 0.001), fissure type (*p* < 0.001), and the side of the tooth (*p* < 0.001) remained significantly associated with the presence of a tooth fracture (Table 2). Horses with a longer follow-up showed higher odds of presenting a tooth fracture (*OR* = 3.37; 95% CI, 1.71–6.66). Partial crown fractures were more frequently observed in mandibular cheek teeth (69.4%, 50/72) compared to maxillary cheek teeth (30.6%, 22/72). The odds for a maxillary cheek tooth to fracture was 0.43 (95% CI, 0.20–0.59) times smaller compared to mandibular cheek teeth whereas the lingual side of a cheek tooth showed 2.56 (95% CI, 1.50–4.34) times higher odds to fracture. Teeth showed 4.75 (95% CI, 2.29–9.87), 11.06 (95% CI, 5.47–22.35), and 14.27 (95% CI, 4.88–41.71) higher odds to fracture when a type 1a, type 1b and type 2 fissure were present, respectively.

**Table 1:** Results of the association analysis of individual variables with the presence of a tooth fracture.

**Tooth fracture**				
**Variable**	**Category**	**Estimate**	**SE**	**95% CI**
**Time of follow-up**	Cont.	1.38	0.37	0.66–2.10
**Pathology**	No pathology	Reference category		
	Periodontitis	0.67	0.66	−0.62–1.97
	Wear disorder	1.56	0.54	0.51–2.62
**Jaw**	Mandible	Reference category		
	Maxilla	−0.86	0.26	−1.37 to −0.34
**Fissure present**	Yes	Reference category		
	No	−1.91	0.33	−2.56 to 1.28
**Fissure type**	No	Reference category		
	1a	1.48	0.37	0.75–2.21
	1b	2.22	0.36	1.53–2.92
	2	2.59	0.55	1.52–3.66

**Table 2:** Final model of all variables associated with a tooth fracture.

**Tooth fracture**				
**Variable**	**Category**	**Estimate**	**SE**	**95% CI**
**Time of follow-up**	Cont.	1.22	0.35	0.53–1.90
**Jaw**	Mandible	Reference category		
	Maxilla	−1.07	0.27	−1.60 to −0.53
**Fissure type**	No	Reference category		
	1a	1.56	0.37	0.83–2.29
	1b	2.40	0.36	1.70–3.11
	2	2.66	0.55	1.58–3.73
**Tooth side**	Buccal	Reference category		
	Lingual	0.94	0.27	0.41–1.47

When inspecting the appearance of fissures prior to fracturing it was observed that all fissures that evolved into a partial crown fracture involved or crossed the outer enamel ring. Fissure characteristics remained unchanged in the majority of these fissures before fracturing (42/58; 72.4%) (Figure 2). Sixteen fissures changed in characteristics before fracturing including 10 becoming longer, 1 becoming shorter, 2 changing their configuration, 1 becoming longer and changed in configuration and 2 changing color (1 darkened and 1 became lighter).

**Figure 4:**
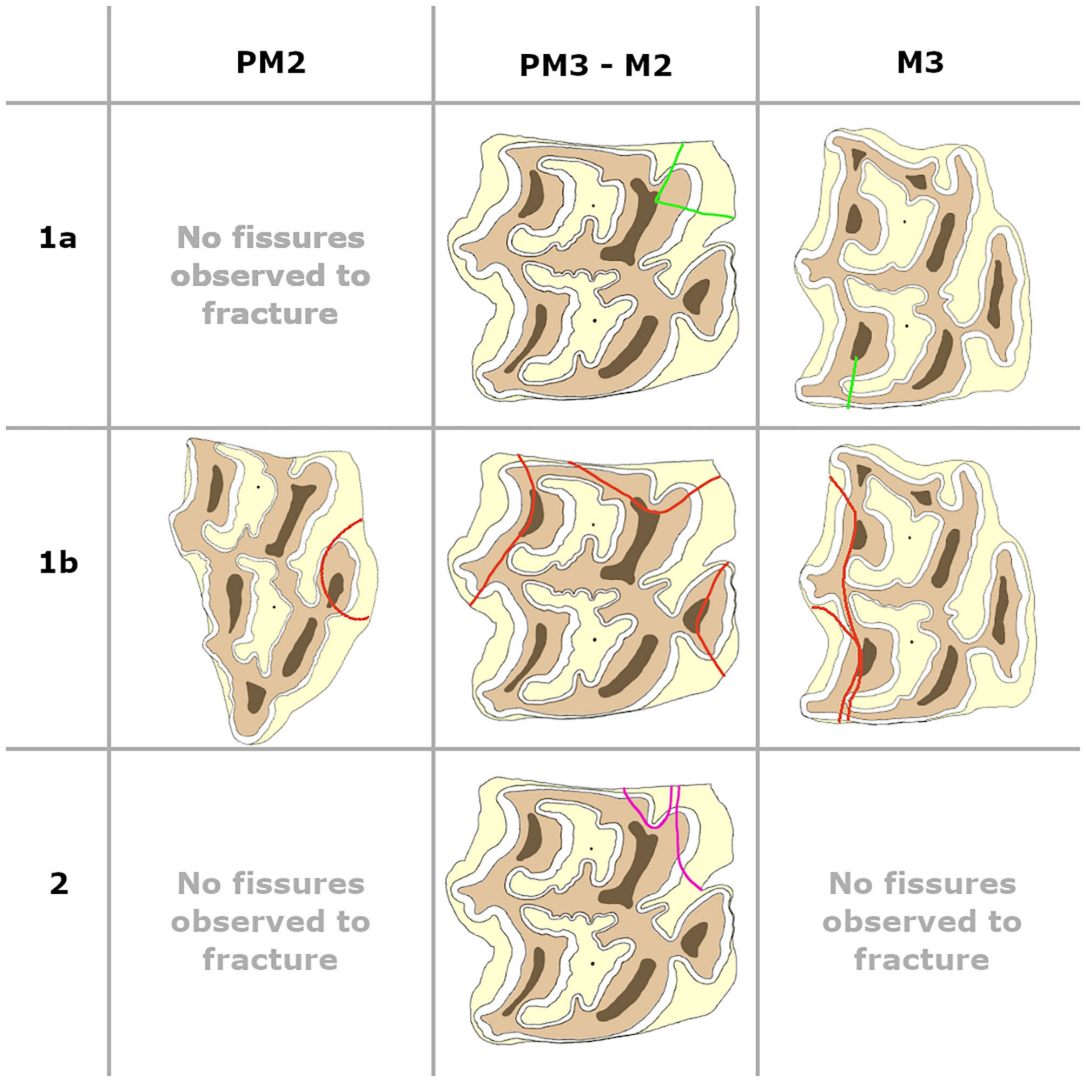
Fissure patterns observed to fracture in maxillary cheek teeth. Left is buccal, right is lingual, top of the picture is distal and the bottom of the picture is mesial.

**Figure 5:**
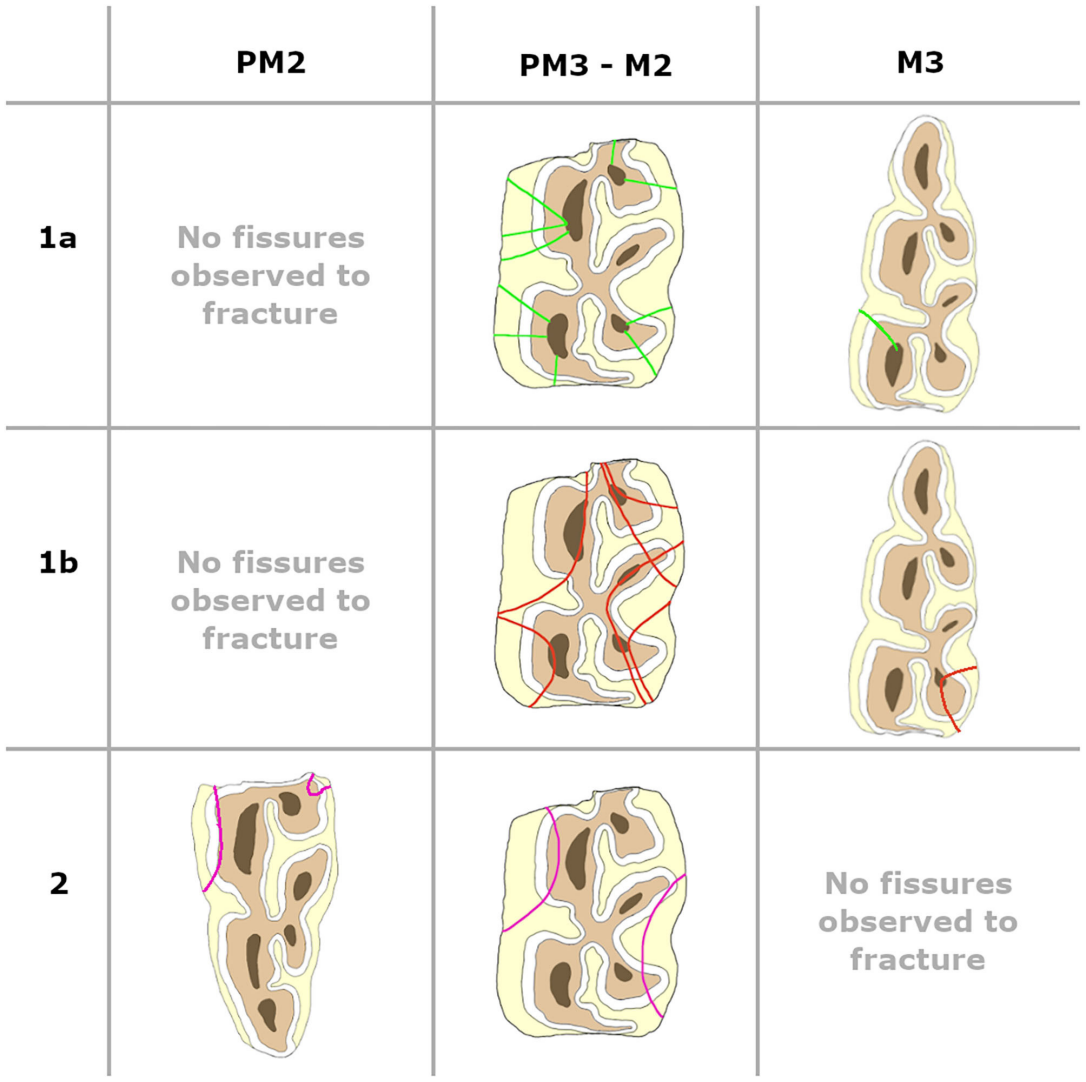
Fissure patterns observed to fracture in mandibular cheek teeth. Left is buccal, right is lingual, top of the picture is distal and the bottom of the picture is mesial.

Fracture patterns varied between fissure types (Figures 4, 5). Fractures evolving from type 1a fissures were more often located in the “corners” of the cheek teeth. A more diverse fracture pattern was recorded when evolving from type 1b fissures. These fractures were mostly located on the lingual side of the tooth (20/29). They were observed on the buccal side of the tooth in 9/29 cases, with one complete buccal slab fracture of a left maxillary third molar tooth (211). Fractures originating from type 2 fissures were recorded on the distolingual side of the tooth in 4/6 cases.

Only one horse developed clinical symptoms (head tilt while eating) related to a crown fracture. In this case, the fractured fragment [buccal slab, left maxillary third molar tooth (211)] was present and caused a lesion in the adjacent mucosa of the cheek. After removal of the fragment the horse showed no further symptoms. The other fractures were only noted at the predetermined time points for follow-up with the fracture fragment already missing. In 13/72 cases, local inflammation of the surrounding gingiva demonstrated the recent nature of those fractures. During the study period, none of the horses developed a complicated fracture characterized by communication with the adjacent pulp cavity.

## Discussion

With the routine use of oral dental scopes by equine dental specialists, small dental anomalies are easily detected but subsequently raises more questions on the clinical importance of these observations. The presence of fine linear defects in the occlusal surface of equine cheek teeth is one of them and it has been demonstrated that these macroscopically visible fissures are in fact true microscopical cracks in the tooth (5, 6). Some authors suggested that occlusal fissures might evolve to crown fractures (3, 4, 7), which is supported by the results of this study (7.4% of fissures evolved to a partial crown fracture in this study population). Equine cheek teeth fractures are major dental lesions and a topic of interest for many researchers. Dental fractures occur in all equine teeth with a low prevalence (0.4–6%) reported in cheek teeth (10, 11). However, this study population demonstrates that horses might develop (uncomplicated) crown fractures more frequently (22/36 horses developed at least one crown fracture). A possible explanation for the relatively high incidence of crown fractures in this population is the set-up of this study with specific attention for crown fractures and using an oral scope in all horses. The observed fractures in this study consisted of low impact partial crown fractures of very limited clinical importance and subsequently remaining without obvious signs of oral discomfort. These fracture types might thus be overlooked during routine dental examinations and not recorded on dental record forms. Therefore, the true prevalence of different cheek tooth fractures might be higher in the general population than previously reported due to a more selective recording of relevant dental pathology.

During the study period, identified fissures were observed to remain unchanged, to disappear and to change in configuration, in length or color demonstrating the dynamics that occur in the occlusal surface of equine cheek teeth due to the continuous grinding action during mastication. Type 1a and type 1b fissures predominantly remained unchanged (69 and 71%, respectively), while this was only the case in 23% of type 2 fissures. Type 2 fissures disappeared in 38% as opposed to only 8% of type 1a and 6% of type 1b fissures. The authors suggest that fissures that became shorter or lighter in color over time were more likely to disappear, which seems particularly true for type 2 fissures where 5/10 fissures that became shorter also disappeared. However, due to the relative low number of observations of fissures becoming lighter (19/785) or shorter (38/785) it is not possible to make definite conclusions on this matter. None of the fissures that became longer during the study disappeared during the follow-up period.

A total number of 72 crown fractures were recorded of which 59 developed at the level where before a fissure had been identified. All fissure types proved to be a significant risk factor for a tooth to develop a crown fracture at that specific location. Furthermore, it was shown that type 2 fissures had the highest odds to evolve into a crown fracture with 13% observed to fracture followed by type 1b (10%) fissures. These findings support previous hypotheses that fissures with patterns in a more mesio-distal plane are more prone to develop into gross crown fractures (3, 4, 6, 7, 12). In contrast, fissures with a largely transverse orientation (type 1a) were also observed to develop into crown fractures (5%). Clinically it remains impossible to predict with certainty which fissures will evolve into a crown fracture. However, it was observed that all fissures that evolved into a crown fracture only involved the marginal ridge(s) of the tooth. It might be suspected that fissures that became darker might be more susceptible to fracture since this darker color might indicate a higher uptake of plant material and thus a wider fissure gap. However, only 1/24 fissure that became darker was observed to fracture afterwards. Similarly, it might also be suspected that fissures that became longer might be more susceptible to fracture since they might further weaken the tooth, which was the case in 11/59 fissures. Unfortunately, due to the relative low number of observations of fissures becoming darker (24/785) or longer (58/785), it is not possible to draw firm conclusions in this matter. Fracture configurations of crown fractures without previously detected fissures showed similar fracture patterns compared to crown fractures that did develop from previously identified fissures (mainly type 1b). Therefore, we hypothesize that they share a similar etiopathogenesis. Several studies have been performed describing the characteristics of the most common fracture planes (8, 10, 13). The most common “idiopathic” cheek teeth fractures are buccal slab fractures in maxillary and mandibular cheek teeth through the secondary dentine of pulp horns 1 and 2. Additional less common maxillary and mandibular miscellaneous fracture patterns have also been observed (8). In the present study population, only one complete buccal slab fracture was observed in a left maxillary third molar (211) tooth, however partial buccal slab fractures (only involving the secondary dentin of pulp horn 1 or 2) were detected more frequently. In maxillary cheek teeth, this type of fracture was one of the most common fracture patterns involving the secondary dentine of pulp horn 2 (4/18). Other fracture types in maxillary cheek teeth were observed at the lingual side which did not match the fracture patterns described by Dacre et al. In contrast to the findings of these authors (8), the most common fracture site in mandibular cheek teeth was located on the lingual side (at the level of pulp horn 3 or 5) (14/41). In this study, mandibular cheek teeth had significant higher odds to develop a tooth fracture, but no predilected teeth was found. This is in contrast with other studies where the maxillary third premolar, first and second molar (triadan 08, 09, 10) and mandibular third premolar and first molar (triadan 08 and 09) teeth were noted to be most commonly involved in crown fractures (8, 10, 13). Cheek teeth in this study were found to have higher odds to fracture on the lingual side of the tooth which again is in contrast with findings of other studies who found that fractures were more frequently found on the buccal side of the tooth (8, 10, 13).

It has been suggested that occlusal fissures are induced by masticatory forces and that due to the interactive mechanical supportive role of the different dentinal tissues, some anatomical locations on the occlusal surface are more prone to mechanical trauma (3, 6). The lingualside of equine maxillary cheek teeth is indeed the position where the masticatory forces are the highest in the maxilla. In contrast, the lingual side of mandibular cheek teeth is not (14). However, when looking at tooth anatomy, there are more enamel infoldings on the lingual side of the mandibular tooth with thinner enamel which might render this side of the tooth weaker (15, 16). The emergence of new fissures in time also supports the suggested theory that fissures are caused by masticatory forces. The presence of dental pathology such as abnormalities of wear was not withheld as a significant risk factor which supports a previous finding that an abnormal wear pattern does not necessarily have a promoting influence on the development of fissures (3). Ramzan and Palmer stated that there was no suggestion that a previous prophylactic dental treatment was a factor in the development of the fissures they observed (2). In the present study, the possible interference of dental treatments on masticatory forces was limited as much as possible by not performing odontoplasty during the study period except when there were signs of clinical discomfort (e.g., oral lesions). This might have had an influence on the occurrence and evolution of detected fissures in the study population.

The aetiopathogenesis of sagittal midline fractures of maxillary cheek teeth has been traced back to severe infundibular caries and are nowadays termed caries-related infundibular fractures (17). In teeth with (idiopathic) fractures that involve pulp horns (e.g., buccal slab fracture), the underlying cause has not been discovered yet. In one study, a reduced dentinal thickness was observed in 25% of these cases indicating that these teeth probably had prior pathological changes with the fracture being a consequence of dental disease rather than a primary pathology (8). Conversely, other studies suggested that fractures involving pulps (including fissures) were the cause for an apical infection in 20% of mandibular cheek teeth and 9% in maxillary cheek teeth (12, 18). It has been shown that many idiopathic fractures do not result in signs of pulpitis or apical disease (4, 10, 13). None of the horses in the present study developed a fracture-related apical infection, and in none of the fractured teeth, a direct communication with the pulp cavity could be found. These findings support the assumption of Dacre et al. that idiopathic crown fractures that communicate with underlying pulp cavities are more likely a secondary pathology of prior dental disease (8).

The clinical sequela most often related with cheek tooth fractures is oral pain caused by mucosal trauma or food pocketing, and periodontitis around the fractured tooth (10, 13). In this study, one horse showed clinical symptoms related to a tooth fracture (head tilt while eating). In this case, the tooth fragment at the level of the fracture (buccal slab, left maxillary third molar [211] tooth) was present causing mucosal trauma of the cheek. After removal of the fragment, the horse showed no further signs of oral discomfort. None of the other horses showed clinical symptoms related to a tooth fracture (2 horses were quidding in the beginning of the study period related to periodontal disease), however it is possible that acute, temporary clinical manifestation of symptoms might have been missed in the horses while they were on pasture. Only in 13/72 cheek tooth fractures, limited local inflammation of the surrounding gingiva was observed in this study population which supports that these partial crown fractures have a low clinical impact. While some equine cheek teeth fractures may result in apical disease (12, 18), more often they do not as is supported by the present study where none of the horses developed a fracture-related apical infection. These results once again reflect the remarkable capability of the equine dentine-pulp complex in walling off the oral environment subsequent to a noxious stimuli.

This research, however, is subject to some limitations. Some horses were prematurely lost during the study period which possibly influenced the outcome of the analysis. The results of this study are furthermore quite population-specific. Nevertheless, the obtained results demonstrate that fissures can evolve into partial crown fractures as we hypothesized. Secondly, during the study period of 3 years none of fractured teeth showed indications of being endodontically compromised. However, a longer follow-up period would have allowed a more extensive evaluation of the risk for pulp disease. Another way to help determine if endodontal disease, such as low grade pulpitis, might have been present was to perform a computed tomography scan.

## Conclusion

The presence of a fissure of any type, mandibular cheek teeth, the lingual side of cheek teeth and time of follow-up proved to be significant risk factors for development of a cheek tooth crown fracture. Type 2 fissures showed the highest odds followed by type 1b fissures. The observed partial crown fractures in this study demonstrated a low clinical impact whereby no tooth developed signs of endodontal disease during the follow-up period.

## Data Availability Statement

The original contributions presented in the study are included in the article/Supplementary Materials, further inquiries can be directed to the corresponding author.

## Ethics Statement

The animal study was reviewed and approved by Ethic committee of the Faculty of Veterinary Medicine Ghent University.

## Author Contributions

LV and EP designed the study. EP performed the execution of the study (dental examinations and processing data), BB analyzed the data. EP, LV, and BB interpreted the findings. EP and LV prepared the manuscript. All co-authors contributed and approved the final version of the manuscript.

## Conflict of Interest

The authors declare that the research was conducted in the absence of any commercial or financial relationships that could be construed as a potential conflict of interest.
