# Supplementary material for: Occlusal Fissures in Equine Cheek Teeth: A Prospective Longitudinal in vivo Study
Source: Front Vet Sci. 2020 Nov 17;7:604420. doi: 10.3389/fvets.2020.604420 (PMC7705111; doi:10.3389/fvets.2020.604420)
Supplement: Supplementary file 1 [file Data_Sheet_1.PDF]

Supplementary information 1.

A) Bar chart illustrating the number of new fissure fractures observed at different time points in the maxilla and the mandible.

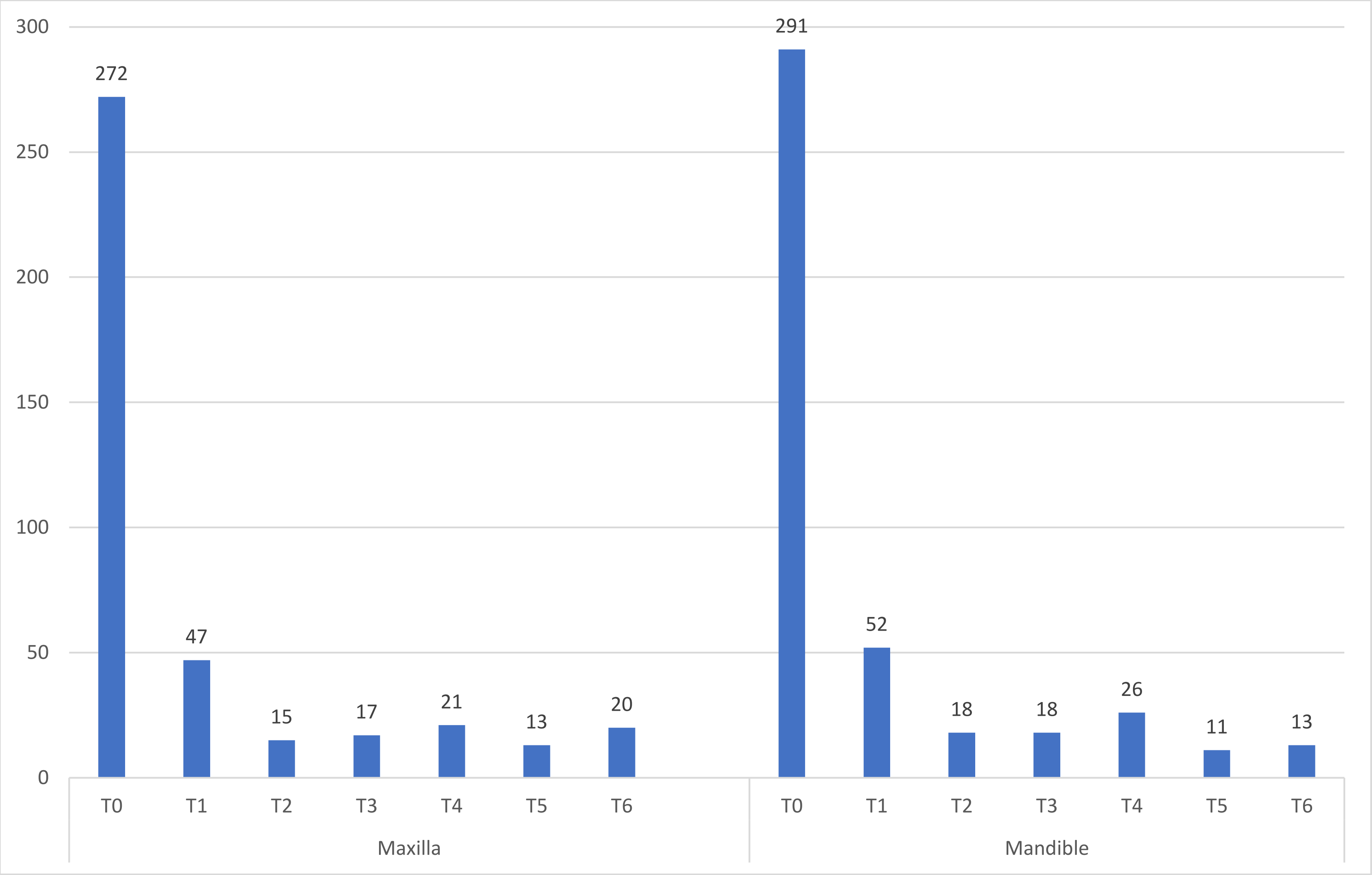

B) Bar chart illustrating the number of new fissure fractures (according to fissure type) observed at different time points in the maxilla and the mandible.

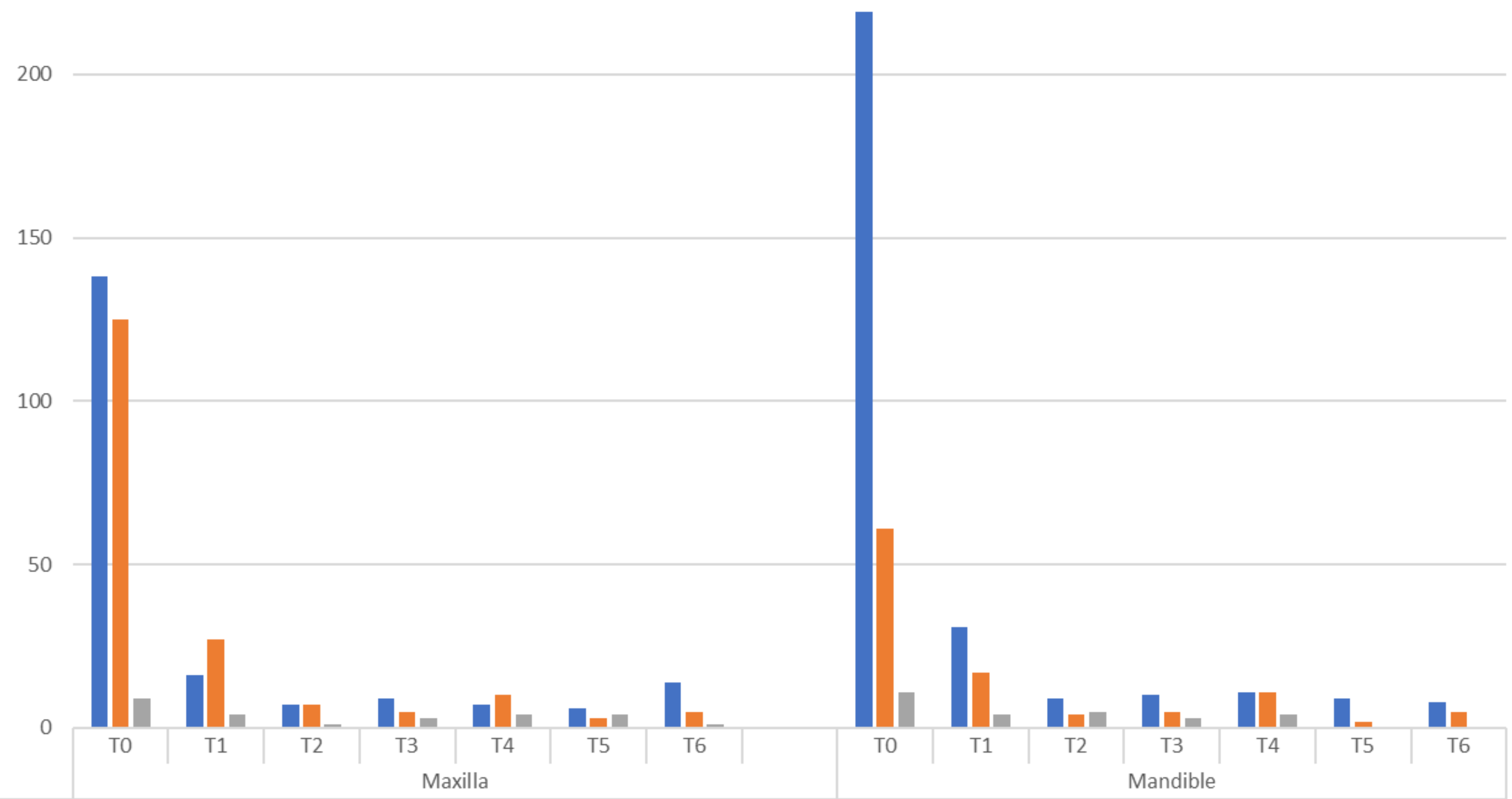

	Maxilla T0	Maxilla T1	Maxilla T2	Maxilla T3	Maxilla T4	Maxilla T5	Maxilla T6	Mandible T0	Mandible T1	Mandible T2	Mandible T3	Mandible T4	Mandible T5	Mandible T6
Type 1a	138	16	7	9	7	6	14	219	31	9	10	11	9	8
Type 1b	125	27	7	5	10	3	5	61	17	4	5	11	2	5
Type 2	9	4	1	3	4	4	1	11	4	5	3	4	0	0
