# Supplementary material for: Occlusal Fissures in Equine Cheek Teeth: A Prospective Longitudinal in vivo Study
Source: Front Vet Sci. 2020 Nov 17;7:604420. doi: 10.3389/fvets.2020.604420 (PMC7705111; doi:10.3389/fvets.2020.604420)

Supplementary information 2. Bar charts illustrating the distribution of fissure fractures and their evolution according to Triadan number in the maxilla and the mandible (fissure fractures where follow-up was not possible are excluded from these bar charts). Top charts are an overview of the evolution of the total number of fissures. Bottom charts illustrate the distribution according to fissure type.

a) Overall distribution of fissure fractures.

Maxilla

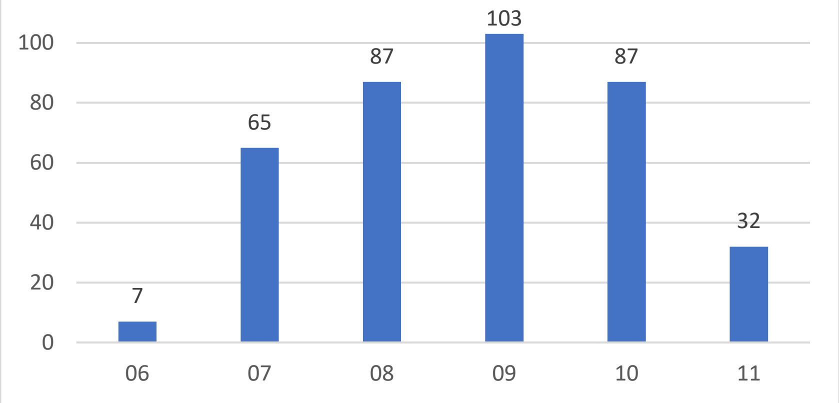

Mandible

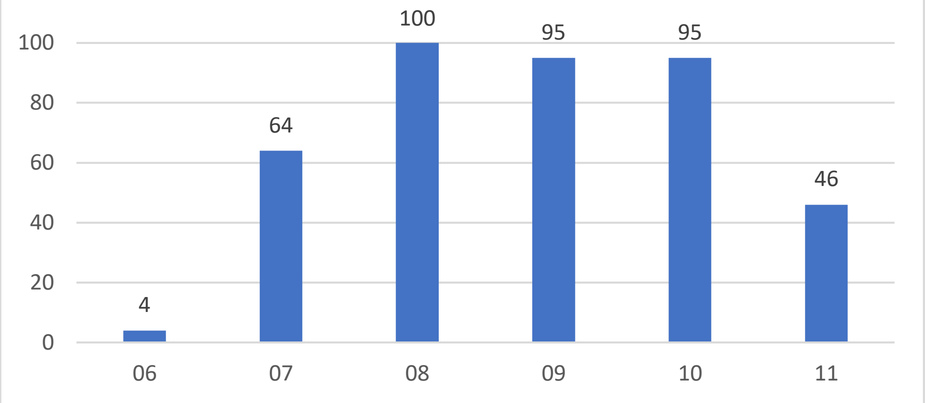

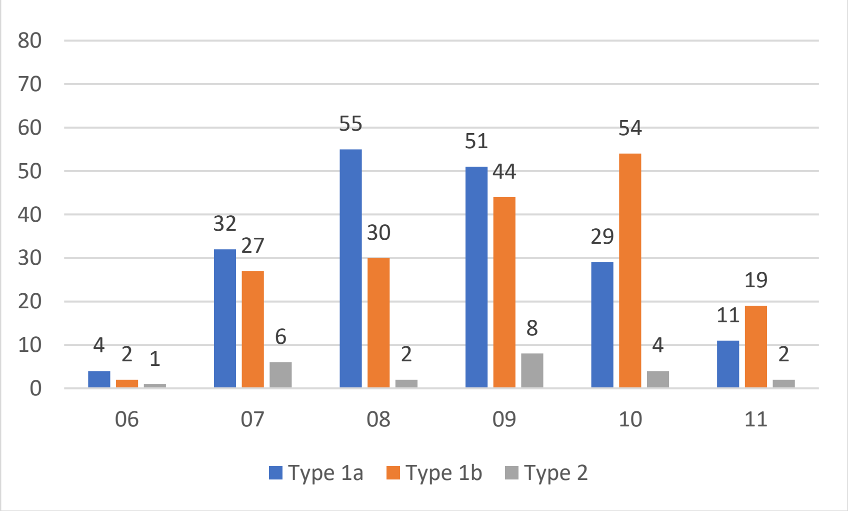

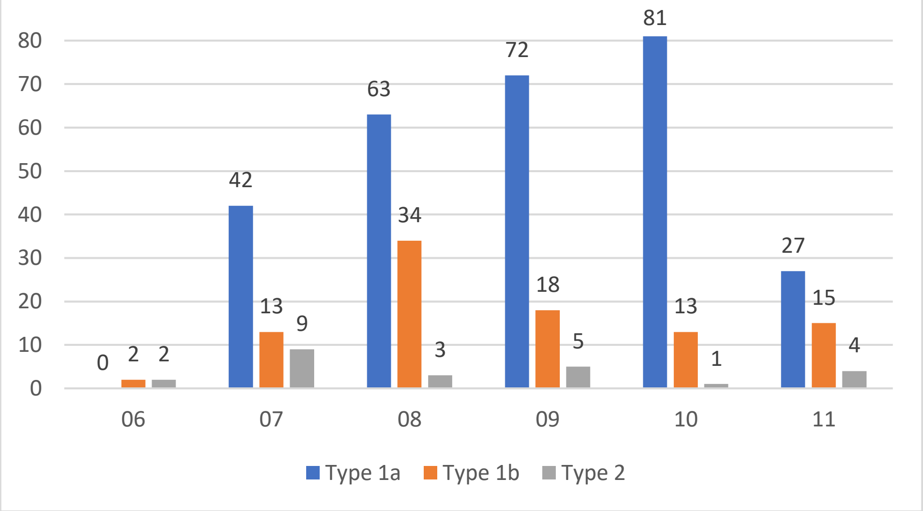

b) Distribution of fissure fracture that remained unchanged.	
Maxilla	Mandible
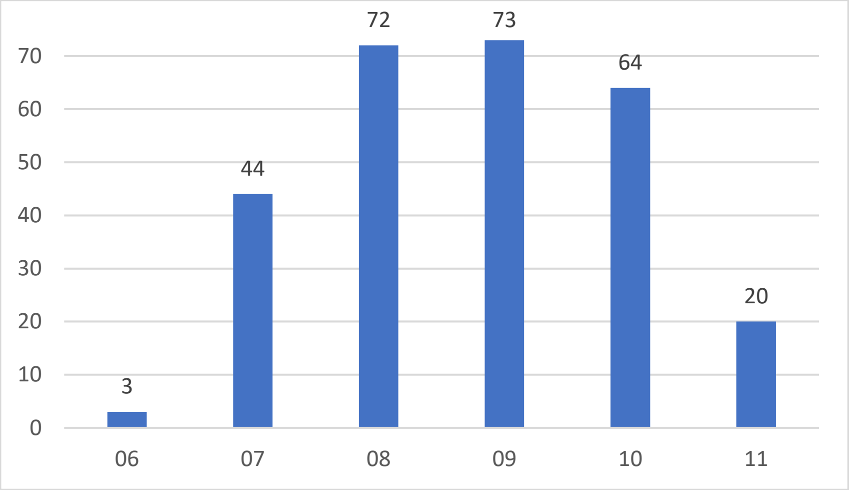	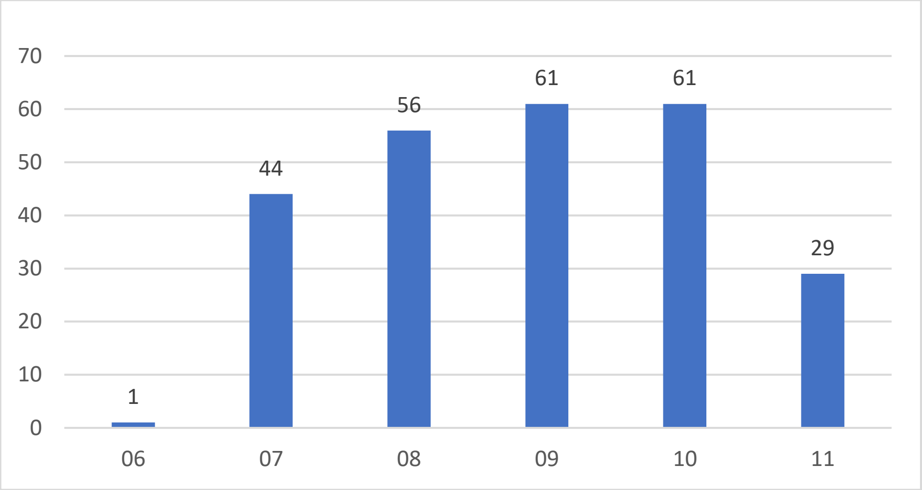
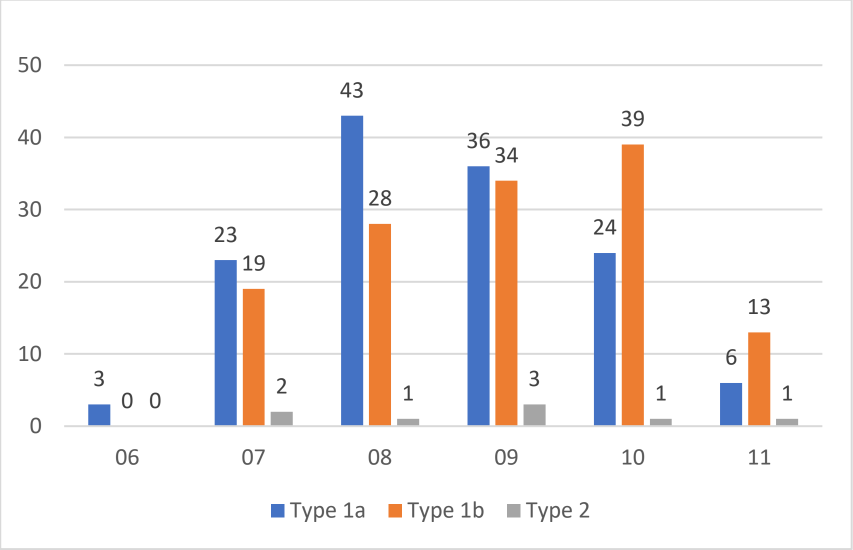	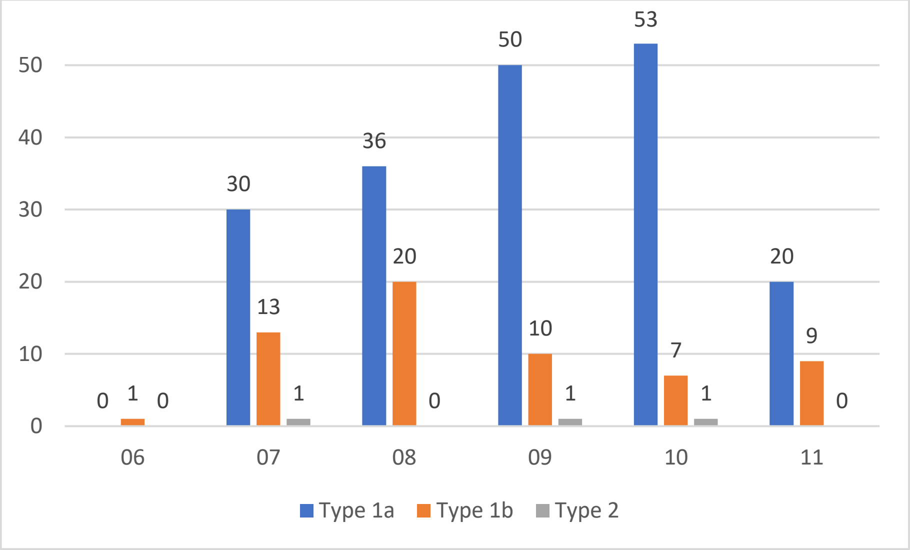

c) Distribution of fissure fractures that disappeared.

Maxilla

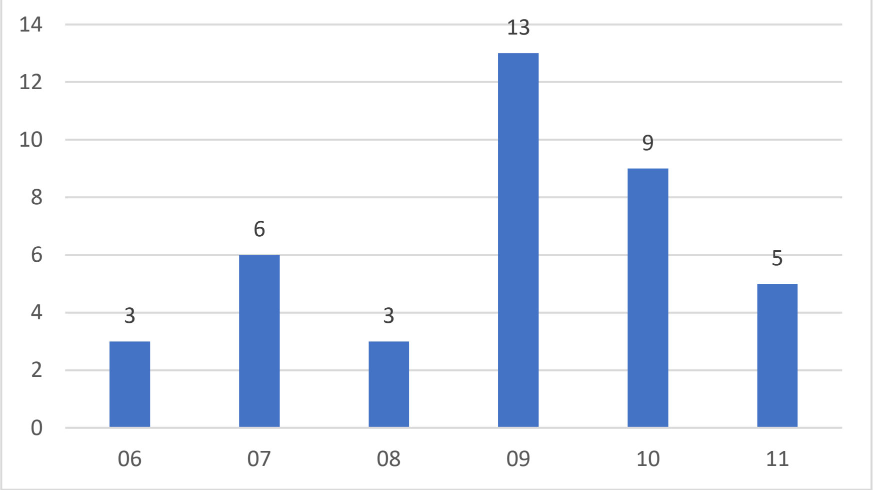

Mandible

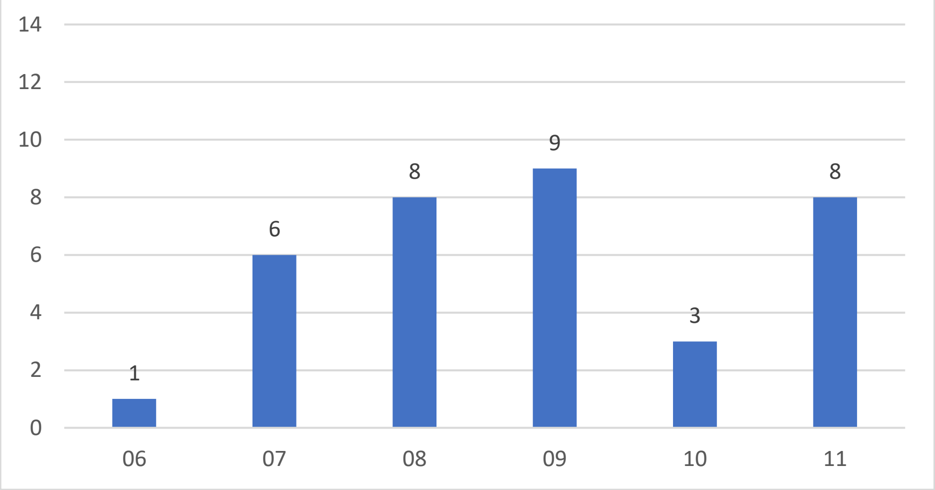

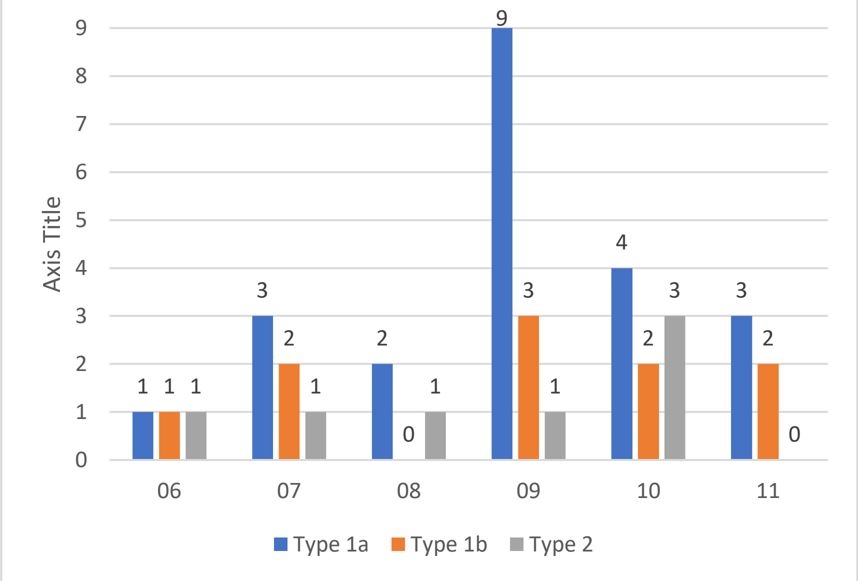

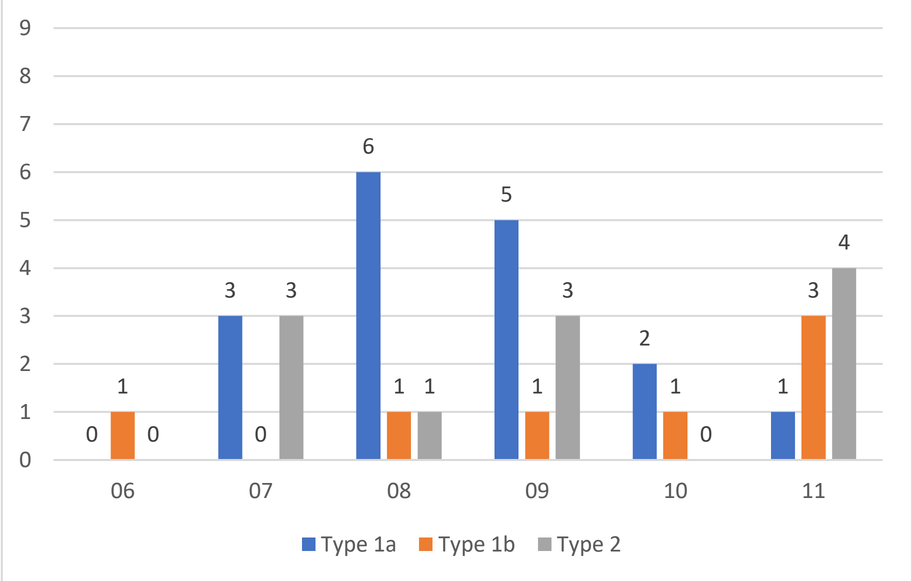

d) Distribution of fissure fractures that changed configuration over time.

Maxilla

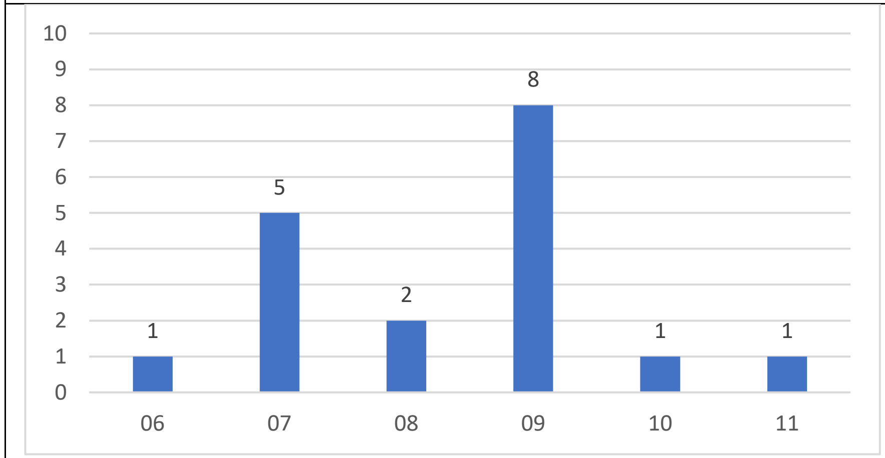

Mandible

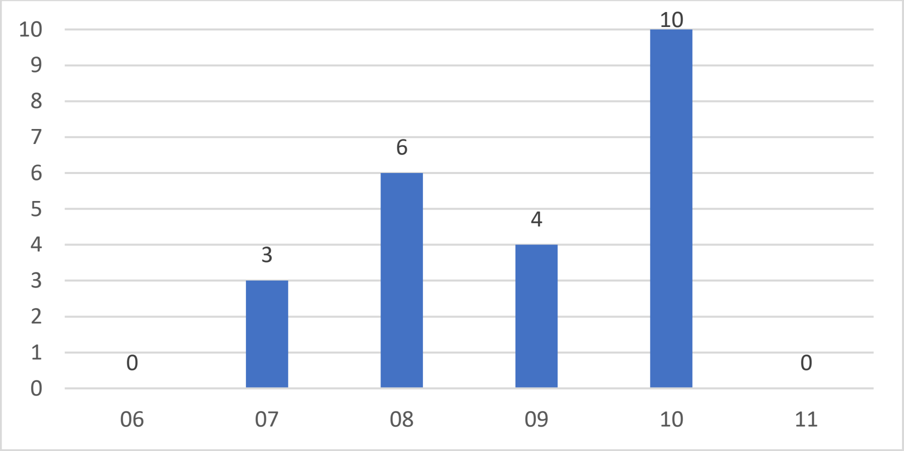

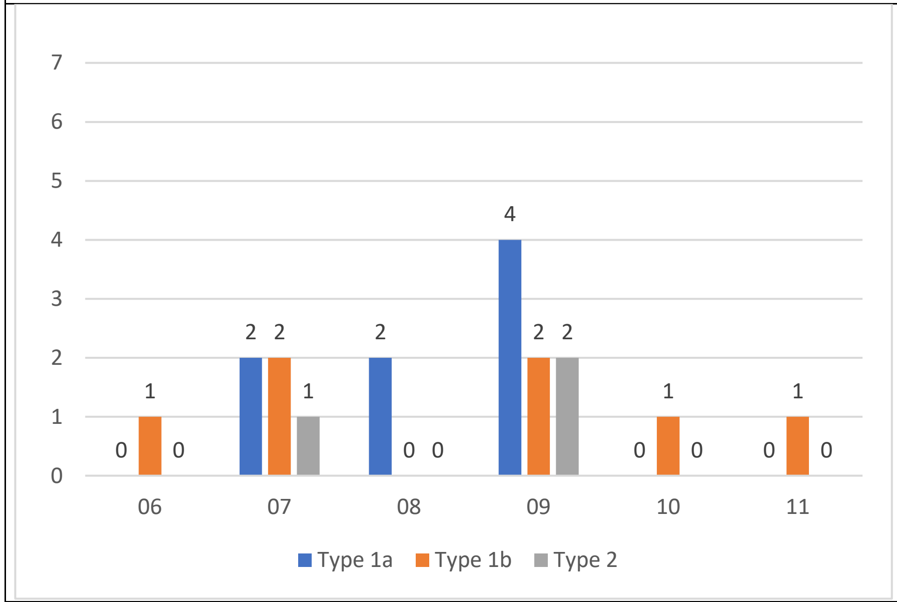

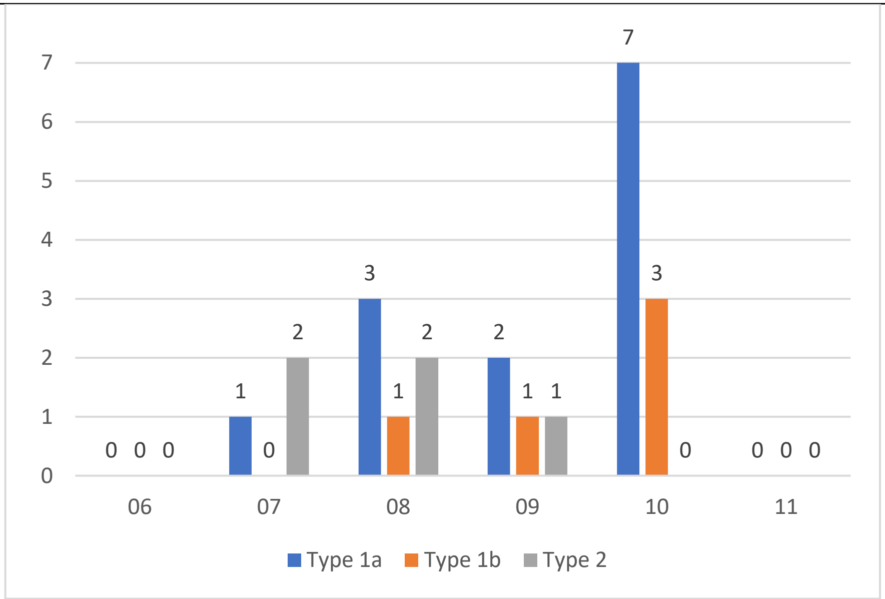

e) Distribution of fissure fractures that became longer.

Maxilla

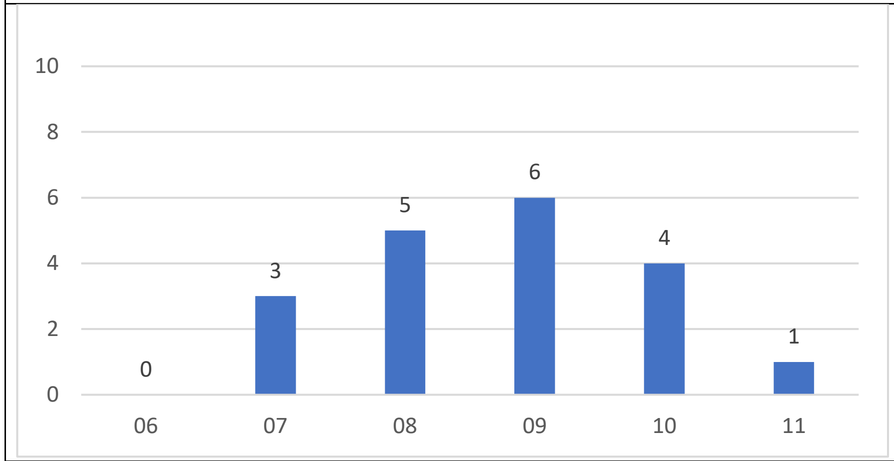

Mandible

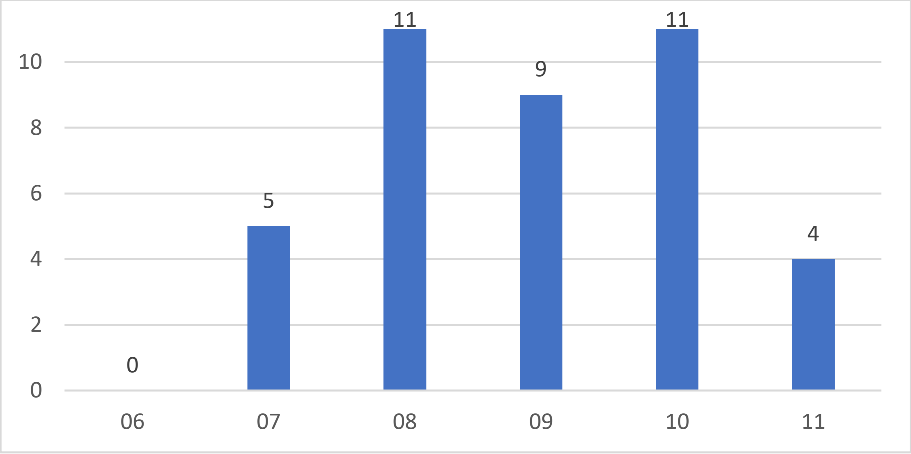

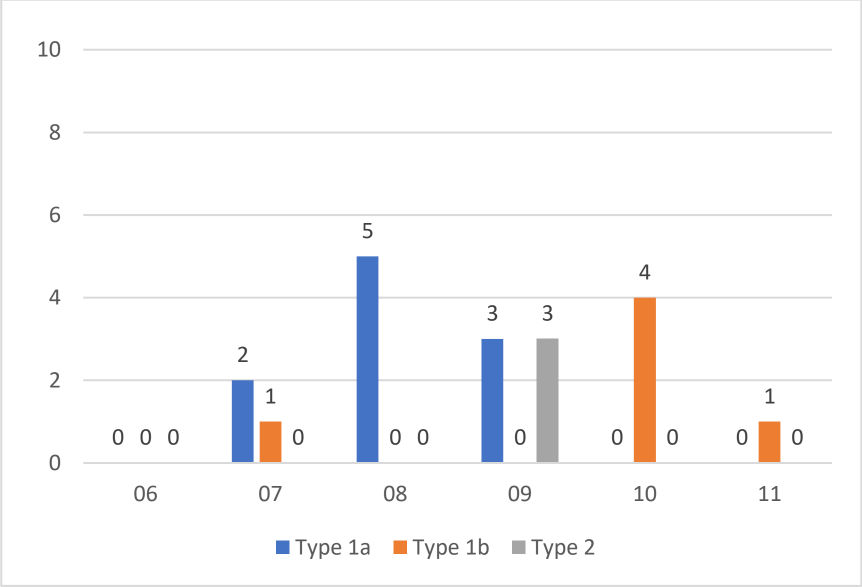

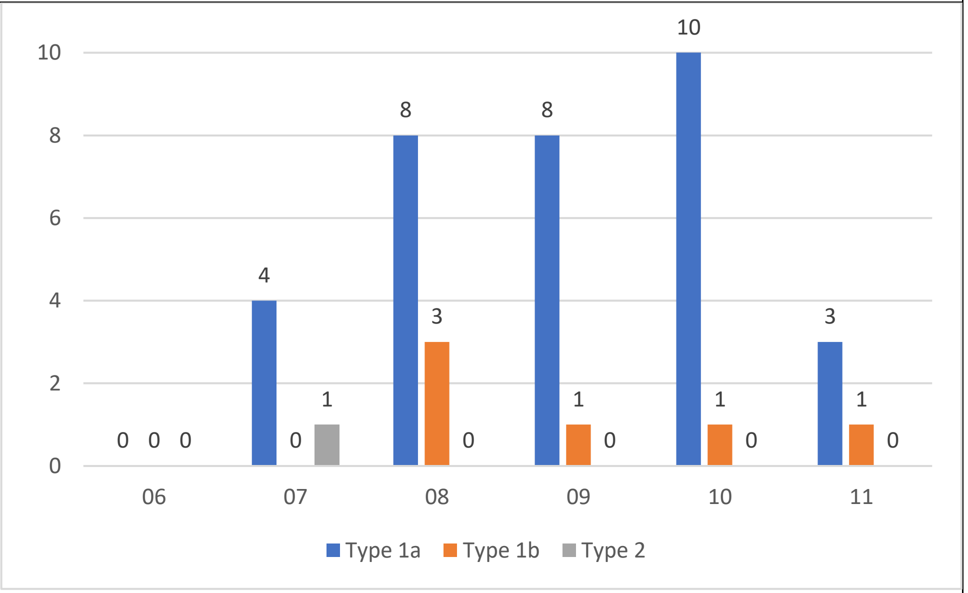

f) Distribution of fissure fractures that became shorter.

Maxilla	Mandible

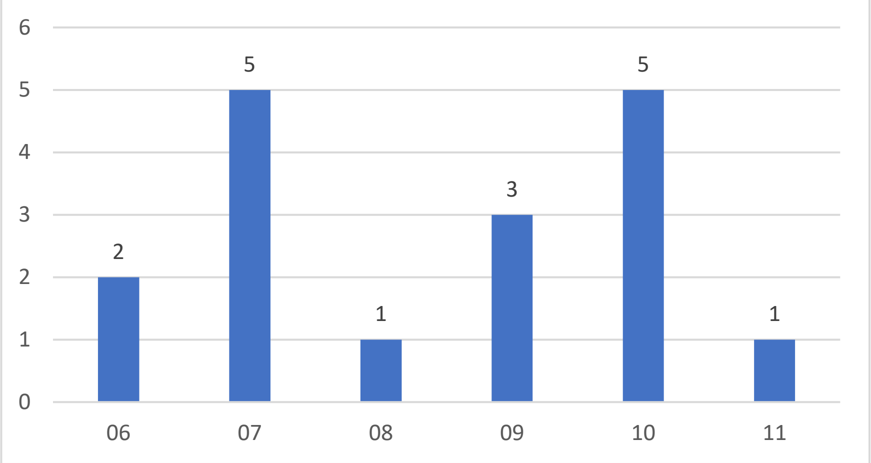

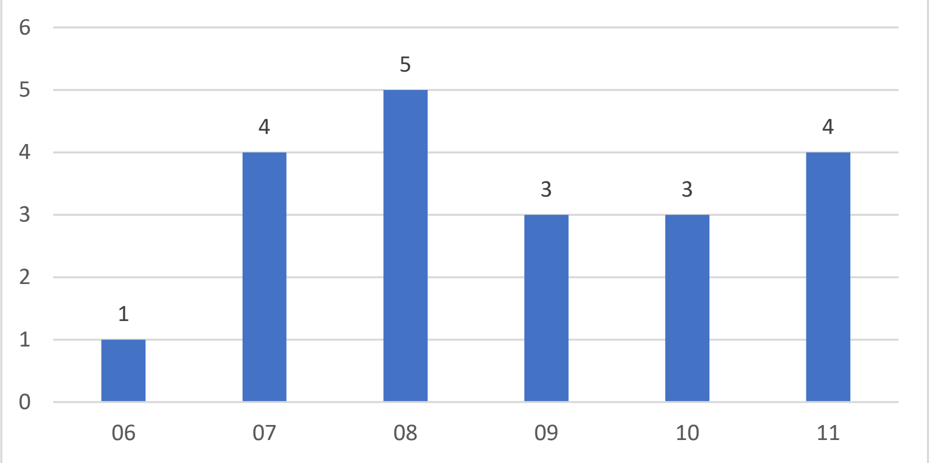

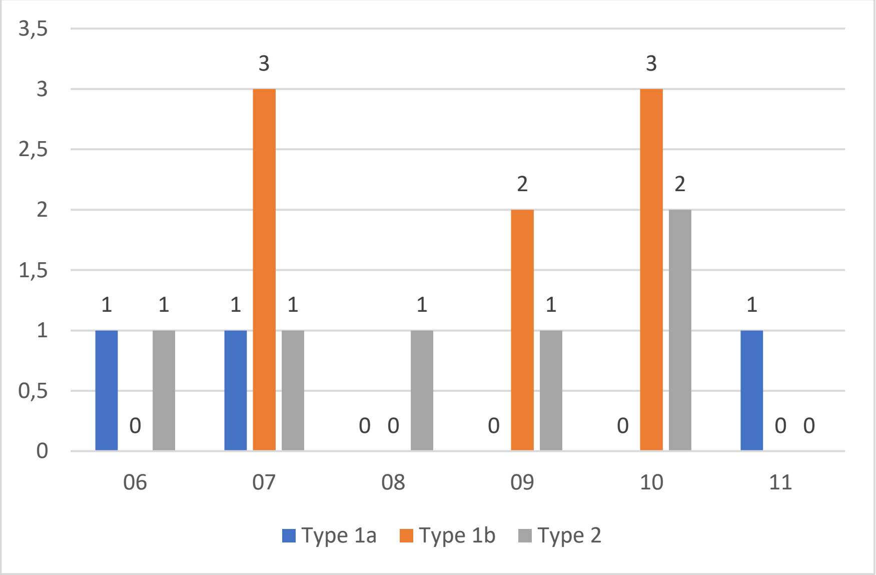

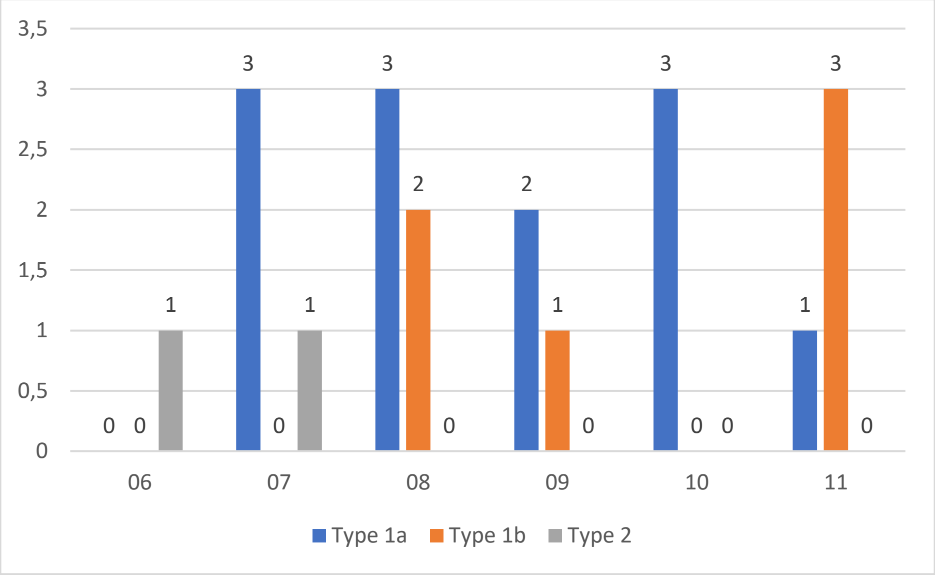

g) Distribution of fissure fractures becoming darker.

Maxilla	Mandible
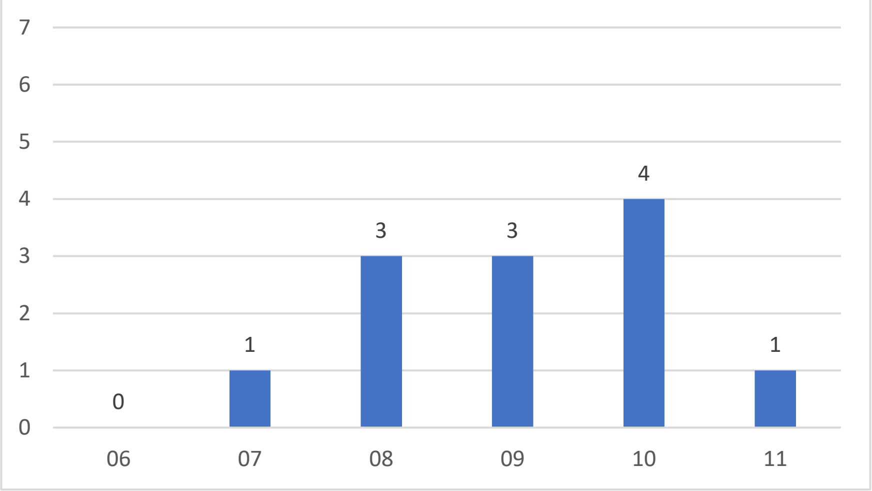	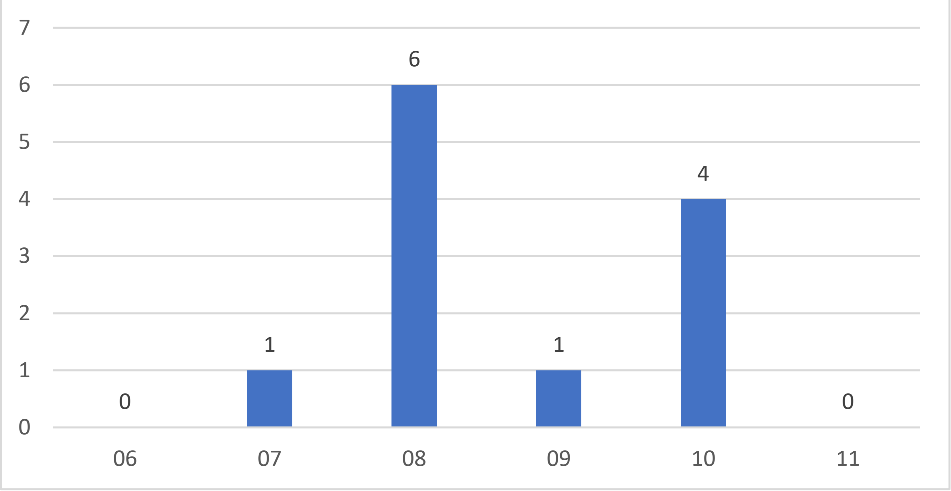
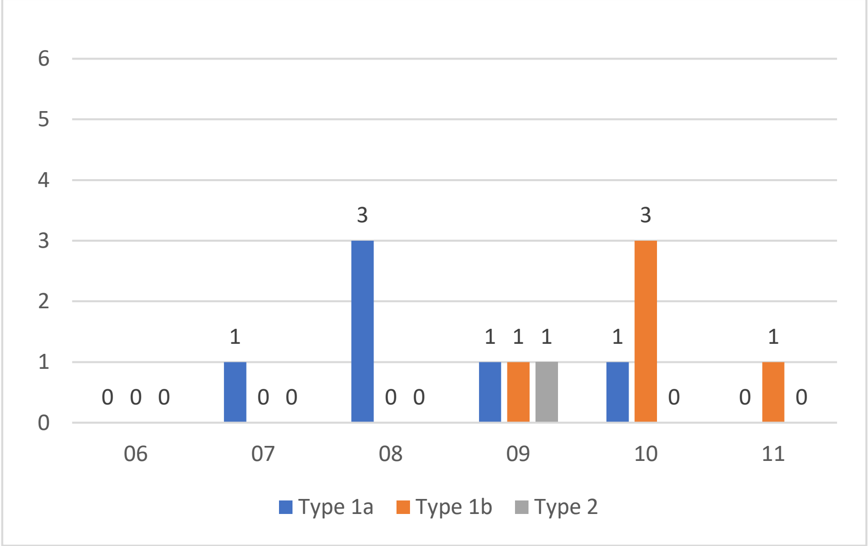	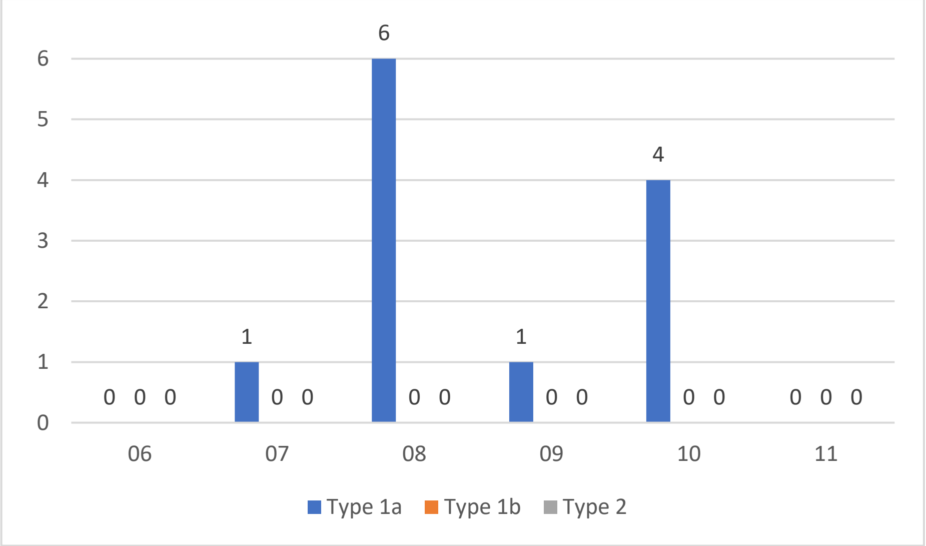

h) Distribution of fissure fractures becoming lighter.

Maxilla	Mandible

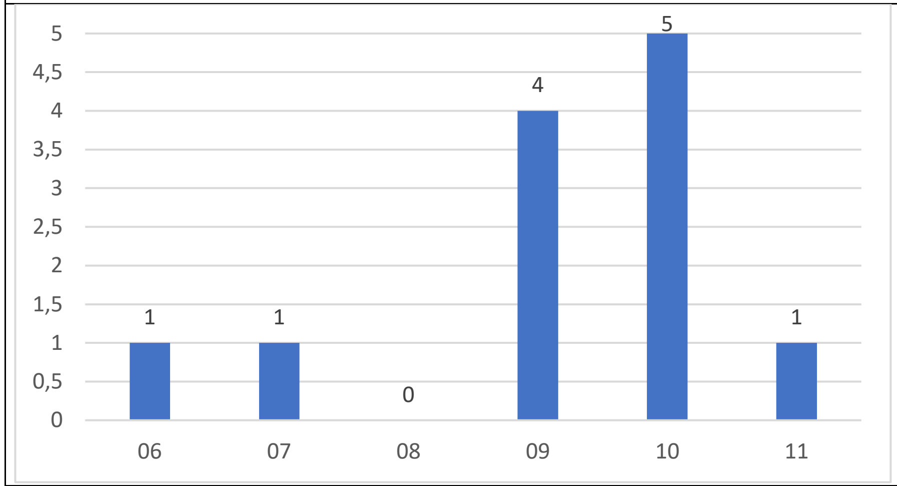

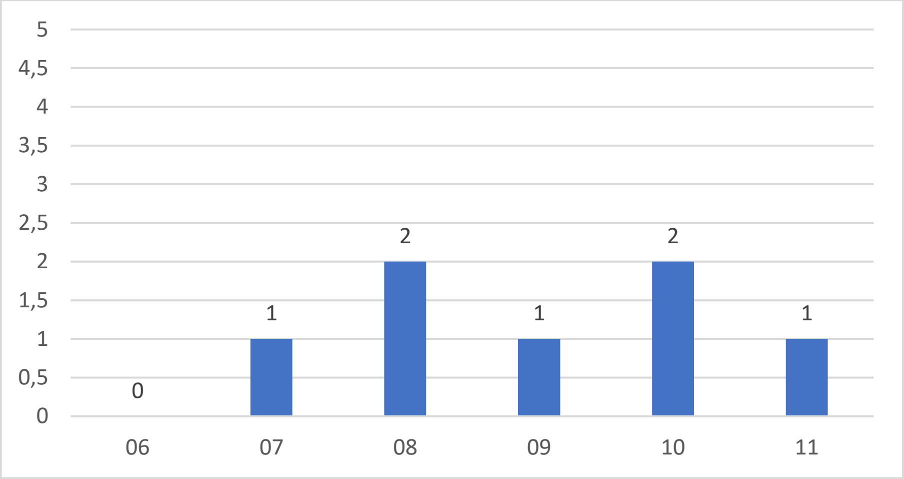

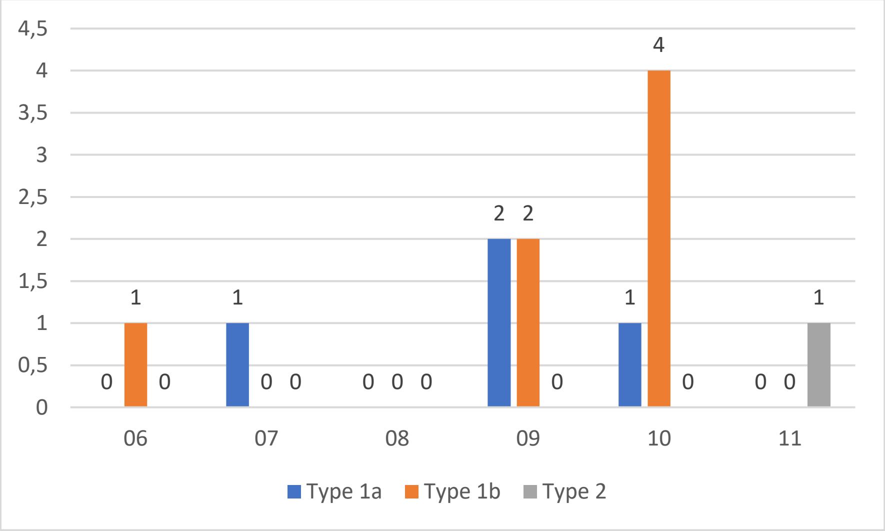

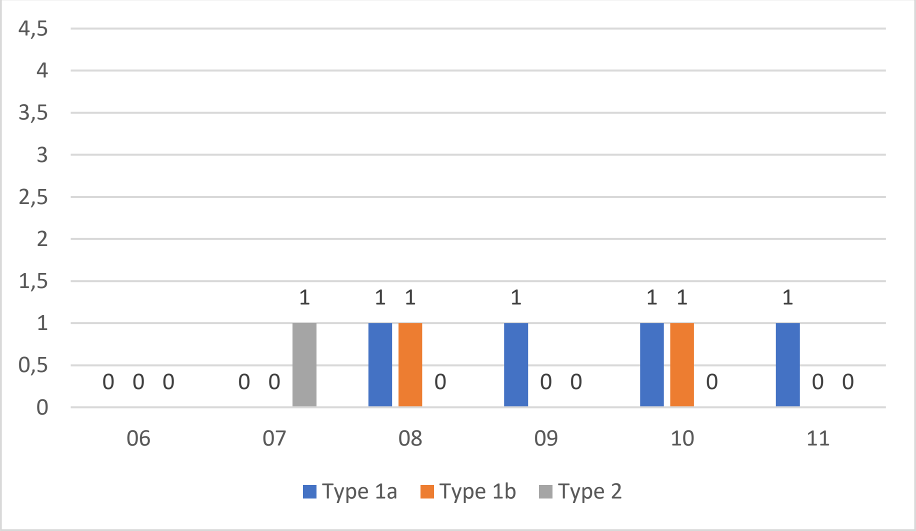

Supplement: Supplementary file 2 [file Data_Sheet_2.PDF]
