# Supplementary figures and images for: Occlusal Fissures in Equine Cheek Teeth: A Prospective Longitudinal in vivo Study
Source: Front Vet Sci. 2020 Nov 17;7:604420. doi: 10.3389/fvets.2020.604420 (PMC7705111; doi:10.3389/fvets.2020.604420)

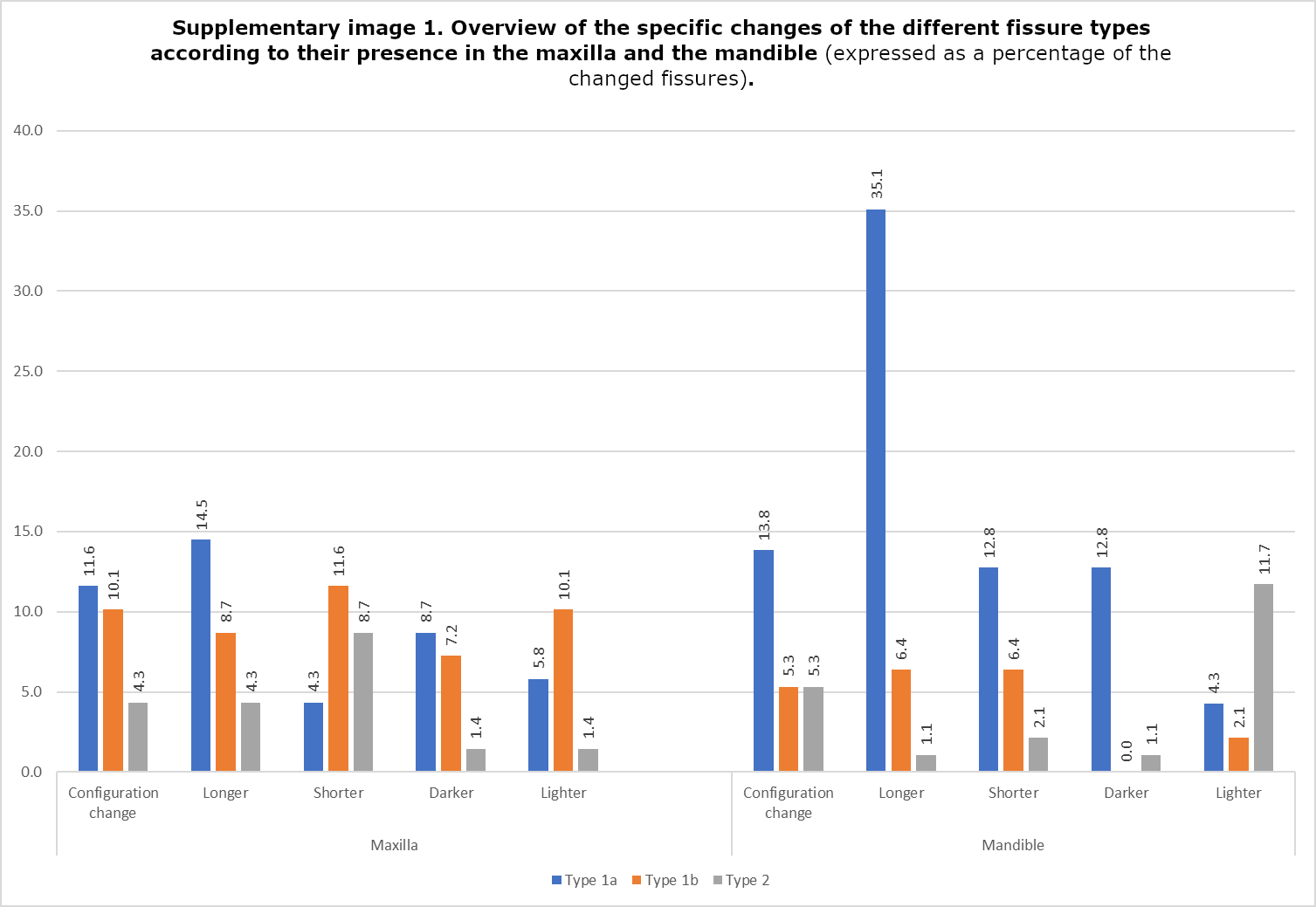

Supplement: Supplementary file 3 [file Image_1.tiff]

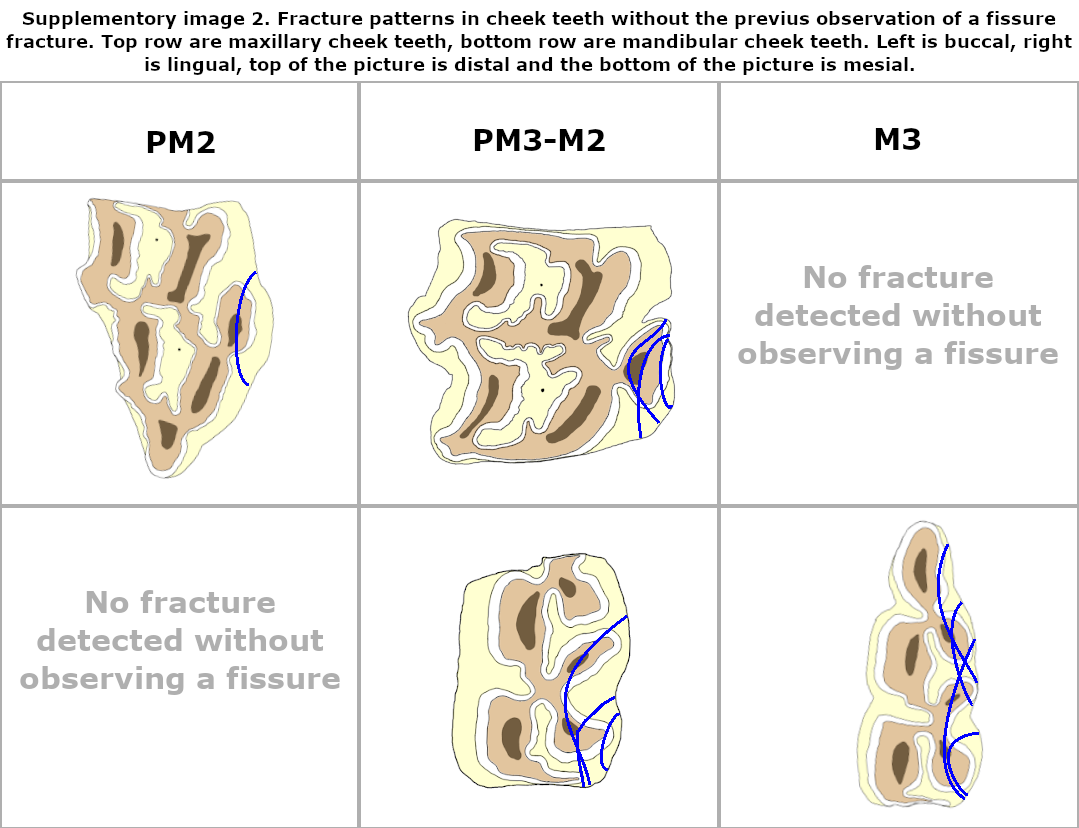

Supplement: Supplementary file 4 [file Image_2.tiff]
